# Dual-flow convolutional neural network for automatic measurement of left ventricular ejection fraction and global longitudinal strain in echocardiography

**DOI:** 10.1371/journal.pdig.0001128

**Published:** 2026-08-28

**Authors:** Zisang Zhang, Ye Zhu, Shujun Chen, Yiheng Dong, Yiwei Zhang, Chun Wu, Ziming Zhang, Shuangshuang Zhu, Manwei Liu, Zhenxing Sun, Peige Zhang, Lili Jiang, Hongliang Yuan, Yongxing Zhang, Yuan Peng, Chunyan Xu, Chunyan Ma, Chunquan Zhang, Xu Huang, Xiaoshan Zhang, Yilu Shi, Xiaocong Wang, Qiaohui Su, Jing Wang, Jing Wang, Mingxing Xie, Xin Yang, Li Zhang, Yuman Li

**Affiliations:** 1 Department of Ultrasound Medicine, Union Hospital, Tongji Medical College, Huazhong University of Science and Technology, Wuhan, China; 2 Clinical Research Center for Medical Imaging in Hubei Province, Wuhan, China; 3 Hubei Province Key Laboratory of Molecular Imaging, Wuhan, China; 4 Media and Communication Lab (MC lab), Electronics and Information Engineering Department, Huazhong University of Science and Technology, Wuhan, China; 5 Department of Cardiovascular Ultrasound, The First Affiliated Hospital of China Medical University, Shenyang, China; 6 Department of Ultrasound Medicine, The Second Affiliated Hospital, Jiangxi Medical College, Nanchang University, Nanchang, China; 7 Department of Ultrasound Medicine, The Affiliated Hospital of Inner Mongolia Medical University, Hohhot, China; 8 Department of Cardiovascular Ultrasound, The First Hospital of Jilin University, Changchun, China; 9 Department of Radiology, Union Hospital, Tongji Medical College, Huazhong University of Science and Technology, Wuhan, China; Weill Cornell Medicine, UNITED STATES OF AMERICA

## Abstract

Left ventricular ejection fraction (LVEF) and global longitudinal strain (GLS) are essential for the diagnosis, clinical decision-making, and prognosis of cardiovascular disease. However, accurate assessments of LVEF and GLS by echocardiography are hampered by inter-observer variability, time-consuming, and labor-intensive. This study aimed to develop an automated method to accurately and rapidly assess LVEF and GLS. Based on the datasets of 500 patients (1,500 videos) from the internal center and 363 patients (1,089 videos) from four external centers, we successfully developed a dual-flow convolutional neural network called Echo-DFCNN, which allowed for synchronous acquisition of LVEF and GLS. We evaluated the performance of the Echo-DFCNN in a cardiac magnetic resonance (CMR) validation dataset composed of 67 patients. On the internal test dataset, the AI and manual measurements of LVEF demonstrated a median absolute error of 3.02% and a mean absolute error of 3.94%. AI-predicted LVEF showed good agreement with manually measured LVEF, with an ICC of 0.927, a bias of 0.89%, and a LOA of -10.91 to 12.69. For GLS, the median absolute error and mean absolute error between AI and manual measurements were 1.43% and 1.83%. AI-predicted GLS exhibited high agreement with manually measured GLS (ICC = 0.913; bias = -1.22%, LOA = -5.12 to 2.68). In addition, Echo-DFCNN maintained good performance when applied to external validation datasets. In the CMR validation dataset, the AI model showed good agreement with CMR measurements for both LVEF and GLS. Echo-DFCNN achieves simultaneous and precise assessment of LVEF and GLS in the study cohorts, demonstrating its potential for robust performance across a wide range of cardiac functions, different image qualities, and machine types.

## Author summary

The assessment of cardiac function is essential for the diagnosis and management of cardiovascular diseases. Echocardiography is one of the preferred imaging methods for evaluating cardiac function. LVEF and GLS are two critical parameters for the assessment of cardiac function. However, current measurement methods are hampered by inter-observer variability, time-consuming and labor-intensive. In this study, we developed an artificial intelligence model called Echo-DFCNN to automatically and simultaneously measure LVEF and GLS. We trained and validated our model on echocardiographic videos from 500 patients at our medical center and 363 patients at four external medical centers. The results demonstrated that the AI measurements were consistent with manual measurements. We further evaluated the performance of the Echo-DFCNN in a CMR validation dataset composed of 67 patients. The results showed that AI measurements achieved good agreement with CMR. This study reveals that our automated approach can accurately and rapidly assess cardiac function, potentially reducing observer variability, improving diagnostic efficiency, and providing support for the diagnosis and management of cardiovascular diseases.

## Introduction

Accurate assessment of left ventricular systolic function is of great value for screening, diagnosis, and therapeutic decision-making, and prognostic evaluation of cardiac disease [1–5]. Echocardiography is considered an essential first-line imaging modality for cardiac function evaluation, providing non-invasive, real-time quantitative assessments of global and regional left ventricular function [1–5].

Left ventricular ejection fraction (LVEF) is the most commonly used parameter of left ventricular performance in clinical practice [1–5]. Echocardiographic LVEF measurements require manual delineation of the endocardial contour in the apical 2-chamber and 4-chamber views, resulting in significant inter-observer variability [6–9]. In addition, global longitudinal strain (GLS), another critical index of left ventricular function, may sensitively detect the subtle changes in left ventricular systolic abnormalities [10,11]. It is an essential indicator in evaluating subclinical cardiac dysfunction, longitudinal monitoring of cardiac toxicity treatments, and providing diagnostic and prognostic information in a variety of cardiovascular diseases [10–14]. However, the measurement of GLS faces two main challenges: First, GLS measurement relies on operator experience to accurately delineate the endocardial borders, resulting in inter- and intra-observer variability. Second, GLS measurement typically depends on commercial software, and differences in implementation across vendors lead to inter-vendor variability [15,16]. Therefore, it is necessary to develop a highly reproducible, accurate, and rapid automated analysis method that can synchronously assess LVEF and GLS, free of vendor and observer variability. It is essential for improving the reliability, reproducibility, and clinical applicability of cardiac function assessments.

Artificial intelligence (AI) algorithms are widely used in cardiac function evaluation in echocardiography [8,17–28]. AI algorithms have powerful data processing and self-learning capabilities, which can extract effective features from images and quickly output the results. It makes up for the shortcomings of traditional ultrasound examinations, which rely on radiologists’ experience and exhibit observer variability [8,9]. A recent study showed that the AI model could segment the left ventricle frame by frame to realize automatic assessments of LVEF when only two frames, end-diastolic (ED) and end-systolic (ES), were labeled [17]. However, the segmentation network ignored the continuous motion information between frames. In addition, some researchers have used optical flow algorithms to fully utilize continuous motion information between frames to track myocardial motion in two-dimensional (2D) echocardiography frame by frame to measure GLS automatically [21–23]. It is worth noting that the optical flow network needs to obtain the endocardial contour of the first frame as the starting position for the optical flow algorithm to track frame by frame. This highlights the complementary strengths of segmentation networks and optical flow algorithms, pointing to the need for an integrated approach that combines both methods to leverage spatial and temporal information effectively.

Therefore, we aimed to design a dual-flow convolutional neural network based on semantic segmentation and optical flow algorithms (Echo-DFCNN) that automatically analyzes echocardiograms to synchronously acquire LVEF and GLS for cardiac function assessment, with the two tasks of segmentation and optical flow functioning as an integrated pipeline.

## Methods

### Ethics statement

The study was approved by the Ethics Committee of Tongji Medical College, Huazhong University of Science and Technology, Wuhan, China. It was conducted in accordance with the ethical principles of the Declaration of Helsinki. The requirement for informed consent was waived.

### Overview of study design

**Fig 1:**
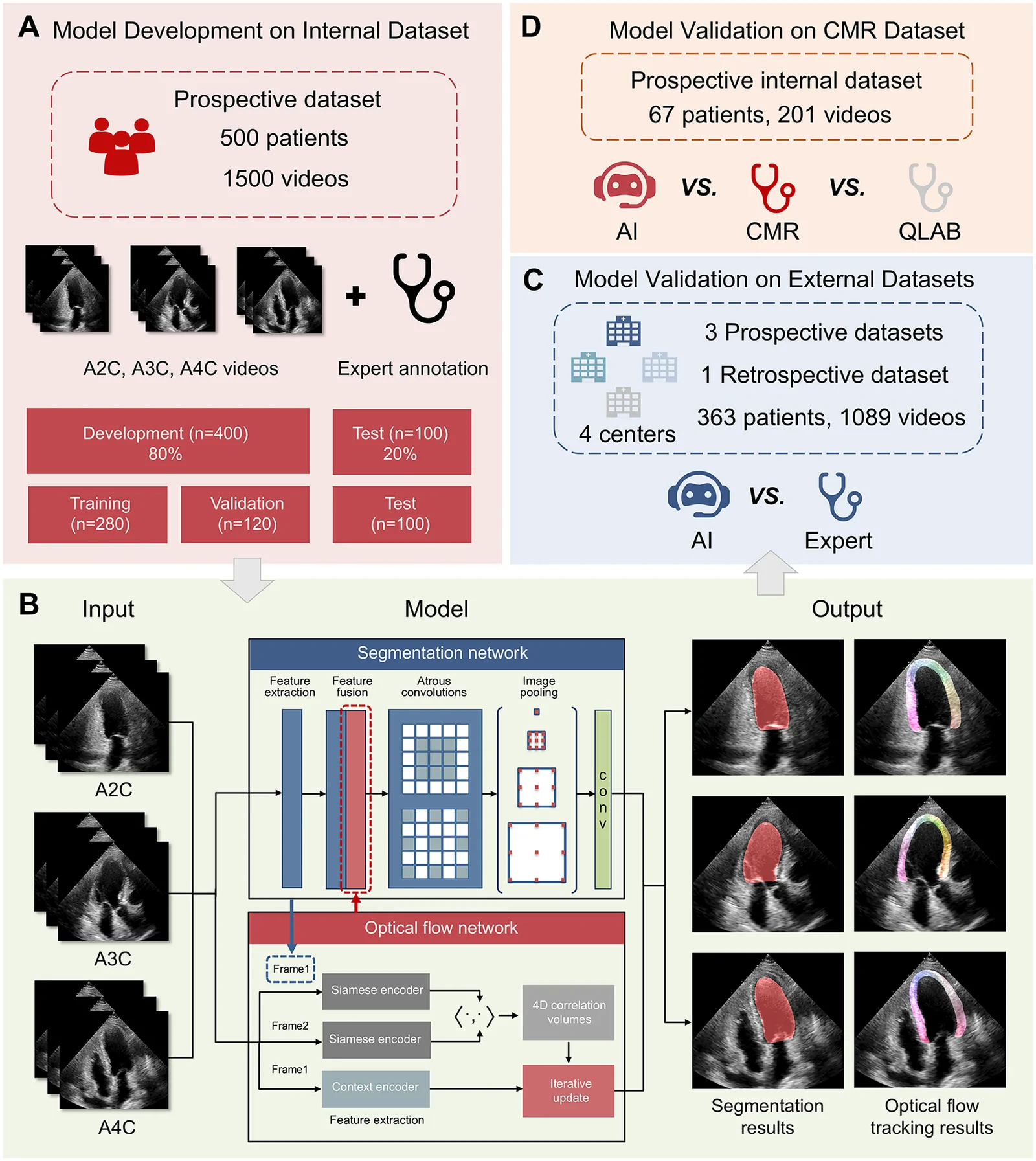
Central illustration. **A,** Based on the internal prospective dataset, we developed a dual-flow convolutional neural network (Echo-DFCNN) to simultaneously analyze the apical 2-chamber, 3-chamber, and 4-chamber echocardiographic videos for LVEF and GLS assessment. **B,** Dual-flow convolutional neural network workflow. **C,** The model was validated on four external datasets to assess its robustness and generalizability. We compared the LVEF and GLS measured by Echo-DFCNN with manual measurements to evaluate the model performance. **D,** The model was validated on the CMR dataset to evaluate its robustness, comparing AI-predicted measurements with CMR measurements (gold standard) as well as Echo-manual measurements (QLAB) with CMR measurements. Specific icons were sourced from open resources: https://openclipart.org/.

In this study, we developed Echo-DFCNN, which analyzes the apical 2-chamber, 3-chamber, and 4-chamber echocardiographic videos to acquire LVEF and GLS simultaneously (Fig 1). We included datasets from five independent centers to ensure the robustness and generalizability of the model. The internal dataset was used for model training, internal validation, and internal testing, and external datasets were used for external validation.

On the internal test dataset, we compared the LVEF and GLS measured by Echo-DFCNN with manual measurements to evaluate the model performance. Further inter-observer and intra-observer reproducibility analyses were performed in this randomized subset of 50 patients from the internal dataset to illustrate the variability of LVEF and GLS measured manually by conventional methods compared to the AI model. For intra-observer variability, the same observers were analyzed again within one month of the first analysis, blinded to previous measurements. For inter-observer variability, measurements were done independently by two observers.

The model performance was compared with the commercially available QLAB Advanced Quantification Software (version 13.0, Philips) on the internal and external datasets to analyze the correlation and agreement between the AI-predicted values and the QLAB values. In order to assess model performance across various cardiac functions, image quality, and machine types, we performed subgroup analyses.

In addition, we included 67 patients from the internal center as the independent cardiac magnetic resonance (CMR) validation cohort and collected both echocardiography and CMR imaging data. The time interval between echocardiography and CMR examinations did not exceed one week. Given this temporal mismatch and the potential for physiological fluctuations in cardiac function during this period, we retrospectively evaluated the clinical stability of the patients between the two examinations. AI-predicted values, QLAB measurements, and CMR measurements were collected, with CMR measurements serving as the gold standard for cardiac function assessment to validate the performance of the AI model.

To further characterize the computational requirements of Echo-DFCNN and provide reference information for future deployment, we evaluated its computational performance in 25 patients randomly selected from the internal test and external datasets under one GPU configuration and three CPU configurations. Two repeats were performed after model loading and warm-up, with the configuration order reversed between repeats to mitigate potential order effects.

### Datasets

Inclusion criteria: 1) ≥18 years old; 2) patients with the standard apical 2-chamber, 3-chamber, and 4-chamber views. Exclusion criteria: 1) Post-processing analysis was not feasible due to insufficient frame rates [29] or file corruption; 2) Incomplete demographic or clinical information. Regardless of the image quality, patients were consecutively enrolled.

This study initially included datasets from five independent medical centers. The internal dataset prospectively included 1536 videos from 512 patients at Wuhan Union Hospital of China from March 2022 to November 2023 for model training, internal validation, and internal testing. Among these, 9 patients were excluded because post-processing analysis with QLAB software was not feasible, and 3 patients were excluded due to incomplete demographic or clinical information. Finally, 1500 videos from 500 patients were included in the internal dataset, which were divided into four subgroups: 1) normal LV systolic function (LVEF≥50%, n = 200); 2) mildly reduced LV systolic function (LVEF = 40% to 49%, n = 100); 3) moderately reduced LV systolic function (LVEF = 30% to 39%, n = 100); 4) severely reduced LV systolic function (LVEF<30%, n = 100). This distribution ensured a study population with diverse cardiac function.

The external validation datasets included data from four centers. The external dataset 1 included 300 videos from 100 patients in the Second Affiliated Hospital of Nanchang University from November 2022 to September 2023, with no exclusions. The external dataset 2 included 450 videos from 150 patients in the First Affiliated Hospital of China Medical University from November 2020 to December 2021, with no exclusions. The external dataset 3 initially included 85 patients in the Affiliated Hospital of Inner Mongolia Medical University from June 2024 to October 2024. 5 patients were excluded because post-processing analysis with QLAB software was not feasible, and 3 patients were excluded due to incomplete demographic or clinical information. Finally, 231 videos from 77 patients were included. The external dataset 4 initially included 40 patients in the First Hospital of Jilin University from June 2024 to August 2024. 3 patients were excluded because post-processing analysis with QLAB software was not feasible, and 1 patient was excluded due to incomplete demographic or clinical information. Finally, 108 videos from 36 patients were included.

**Fig 2:**
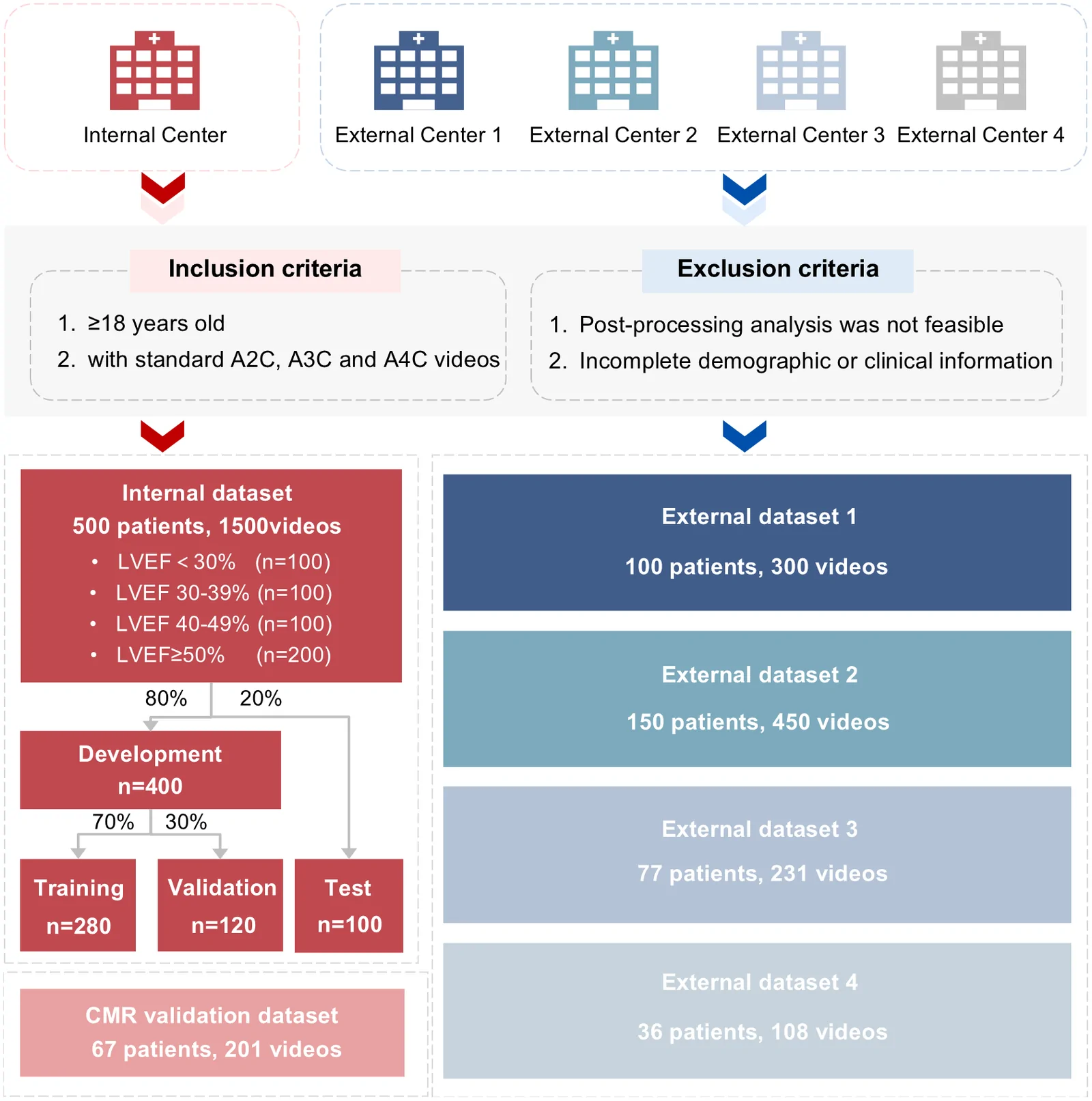
Flowchart of participant inclusion. n, number of patients. Specific icons were sourced from open resources: https://openclipart.org/.

The CMR validation dataset initially included 70 patients at Wuhan Union Hospital of China from September 2017 to September 2023. 3 patients were excluded because post-processing analysis with QLAB software was not feasible. Finally, 201 videos from 67 patients were included. Details of the datasets are provided in Fig 2.

### Echocardiographic examinations

Echocardiographic examinations of the internal dataset were recorded using commercial ultrasound systems (EPIQ 5, EPIQ 7, EPIQ 7C, iE33, or iE Elite systems, Philips). The external datasets included a wider range of ultrasound acquisition systems (LISENDO 880 system, FUJIFILM; EPIQ 5, EPIQ 5C, EPIQ 7C, EPIQ CVx, iE33, iE Elite, iU Elite or iU22 systems, Philips; Vivid E9 or Vivid E95 systems, GE Vingmed Ultrasound). All echocardiographic examinations were conducted in accordance with American Society of Echocardiography (ASE) guidelines [6,7]. LVEF was calculated by biplane Simpson’s method based on the apical 2- and 4- chamber views [6]. GLS was defined as the average peak strain from the three standard apical views (apical 2, 3, and 4-chamber views) [10].

To minimize subjective bias, image quality was blindly and independently evaluated by three radiologists. Each left ventricular segment was assigned a binary visibility score (1 point for clear, 0 for unclear). Specifically, for LVEF measurement, 24 segments were assessed from the apical 2- and 4-chamber views (12 each at end-diastole and end-systole), yielding a maximum score of 24. For GLS measurement, 36 segments were evaluated from the apical 2-, 3-, and 4-chamber views (18 each at end-diastole and end-systole), resulting in a maximum score of 36.

The final continuous quality score for each patient was calculated as the average of the three raters’ scores, rounded to the nearest integer. Inter- observer reliability was assessed using the intraclass correlation coefficient (ICC). To ensure objective categorization, these final scores were divided into tertiles to define three quality subgroups: poor (LVEF: 0–8; GLS: 0–12), fair (LVEF: 9–16; GLS: 13–24), and good (LVEF: 17–24; GLS: 25–36). To verify the robustness of these subgroup categorizations, a bidirectional sensitivity analysis was conducted by shifting the primary cut-offs by ±1 point.

### Model

We developed a dual-flow convolutional neural network based on the semantic segmentation and optical flow algorithms called Echo-DFCNN consisting of two modules. The first module was based on the RAFT architecture [30], automatically tracking left ventricular myocardium motion and calculating GLS. In this module, the segmentation network generated the initial endocardial contour in the key frame, which served as the semantic starting point for subsequent optical flow-based temporal propagation and tracking across the sequence. The second module was based on the Deeplabv3+ [31] architecture to achieve automatic segmentation of the left ventricle and calculate LVEF. In this module, optical flow was utilized to align intermediate frames with the key frames, providing interframe myocardial motion information as temporal context for the segmentation network.

Echo-DFCNN adopted a two-stage training strategy. In the first stage, the optical flow module was trained independently. The ground truth segmentation mask of the starting frame in a sequence was sequentially warped to the ending frame through the predicted optical flows between consecutive frames. A cross-frame segmentation consistency loss was then computed between the warped mask and the ground truth mask of the ending frame. This design enabled the optical flow module to learn cardiac motion patterns without requiring ground truth optical flow annotations, and equipped it with cross-frame alignment capability tailored for echocardiographic sequences. The loss function for this stage is formulated as:


$$\begin{array}{c} L_{flow}=\;L_{seg}\left({\hat{M}}_{warp},M_{gt}\right)+\lambda \left[L_{seg}\left({\hat{M}}_{warp_{in}},M_{in}\right)+L_{seg}({\hat{M}}_{warp_{out}},M_{out})\right] \end{array}$$

where λ is a weight coefficient set to 0.05 in our experiments to balance the main region and boundary constraints.

In the second stage, the segmentation module was trained. The pre-trained optical flow module was frozen. The keyframe and its k neighboring frames were aligned via the frozen optical flow module and then fed into the segmentation module to predict the keyframe’s segmentation map. The loss function for this stage is:


$$\begin{array}{c} L_{segmentation}=\;L_{CE}\left({\hat{P}}_{key},Y_{key}\right)+L_{Dice}\left({\hat{P}}_{key},Y_{key}\right) \end{array}$$

This staged design ensured that the optical flow module first acquired stable cross-frame alignment capability before the segmentation module leveraged the aligned multi-frame features for prediction.

To enhance generalization, data augmentation was integrated into both training stages. Input sequences underwent random scaling and stretching (probability 0.8), random horizontal and vertical flipping (probabilities 0.5 and 0.1, respectively), and random cropping to a fixed resolution of 512 × 512 pixels. A symmetric ColorJitter (probability 0.8) was applied across the entire sequence to maintain temporal photometric consistency. All spatial augmentations were simultaneously applied to the image sequences and corresponding masks to preserve temporal consistency for motion estimation. During segmentation training, an additional independent per-frame ColorJitter (probability 0.2) was applied after the symmetric augmentation to further improve robustness to inter-frame photometric variations.

**Fig 3:**
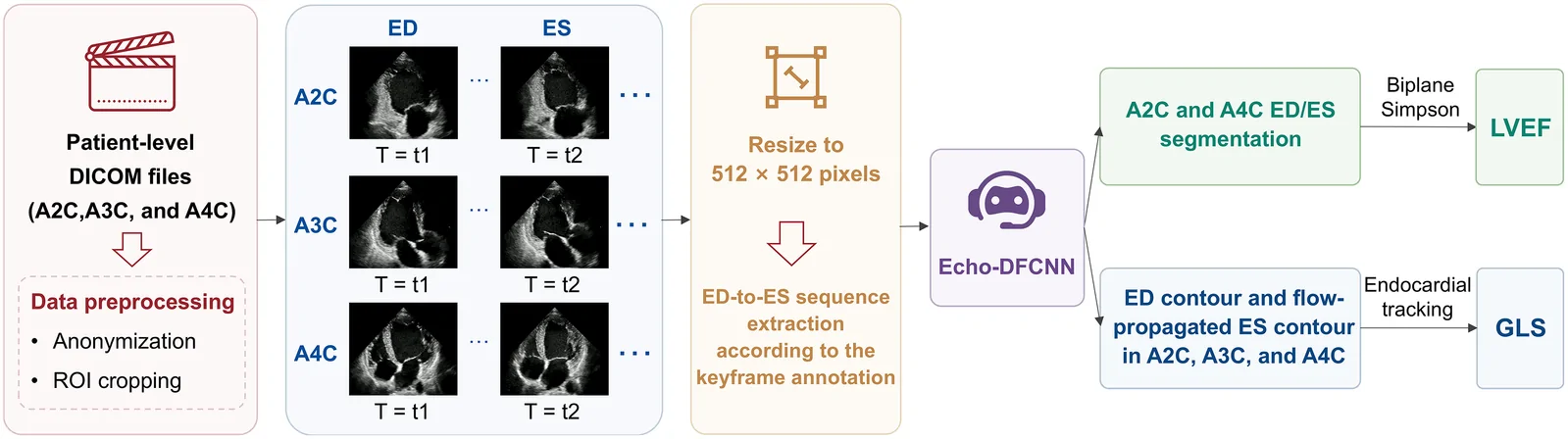
Inference pipeline of Echo-DFCNN. Specific icons were sourced from open resources: https://openclipart.org/.

Model training was performed on two NVIDIA GeForce RTX 3090 GPUs. Detailed training configurations are shown in Table 1. The model architecture is detailed in Supplemental Methods in S1 Appendix. The inference pipeline of Echo-DFCNN is illustrated in Fig 3.

**Table 1:** Optimization configurations for the optical flow and segmentation modules.

	Stage 1: Optical Flow	Stage 2: Segmentation
Total steps	30,000	30,000
Batch size	2	4
Optimizer	AdamW	Adam
Initial learning rate	2 × 10 ^− 5^	2 × 10 ^− 5^
Weight decay	5 × 10 ^− 5^	5 × 10 ^− 5^
LR schedule	MultiStep (×0.5 at steps 2k, 5k, 8k, 15k)	MultiStep (×0.1 at steps 4k, 7k, 10k, 15k, 20k)
Gradient clipping	1.0	1.0

LR, learning rate. The suffix k denotes thousands. MultiStep refers to the learning rate scheduling strategy, where the current learning rate is multiplied by a specific decay factor at the predefined step milestones.

### Statistical analysis

The Kolmogorov-Smirnov test was used to test for the normality of data [32]. Continuous variables for normally distributed data are represented as means and standard deviations, while those for non-normally distributed data are represented as medians and interquartile ranges. Categorical variables are expressed as frequency (percentage).

The accuracy of left ventricular segmentation was evaluated by the Dice similarity coefficient (DSC) and average surface distance (ASD) [33] (Supplemental Methods in S1 Appendix). The accuracy of optical flow tracking of endocardial contours was evaluated by ASD and Hausdorff distance (HD) [33] (Supplemental Methods in S1 Appendix). Pearson or Spearman correlation coefficients were used to assess the correlations for normal or non-normal data, respectively [34]. The differences in correlation coefficients were compared using MedCalc software. Agreement between different measurements was assessed by Bland-Altman analysis and ICC (two-way random-effects model, absolute-agreement definition) [35,36]. Bias (mean difference), limits of agreement (LOA), and standard deviation of bias (SD of bias) were calculated.

The Kruskal-Wallis H test was applied to compare LVEF and GLS measurement errors among subgroups [37]. The p-values from all these Kruskal-Wallis H tests were systematically corrected using the Benjamini-Hochberg False Discovery Rate (FDR) method [38]. When the overall Kruskal-Wallis H-test showed statistical significance (adjusted p < 0.05), pairwise comparisons were conducted using the Mann-Whitney U test [37]. The p-values from all these pairwise comparisons were also systematically corrected using the Benjamini-Hochberg method [38]. To compare the LVEF measurement errors between the Assisted-seg and Single-seg methods, the Wilcoxon signed-rank test for paired samples was applied, and the p-values were corrected using the Benjamini-Hochberg method [38,39]. All statistical analyses were performed using Python version 3.7 and MedCalc version 18.2.1 (MedCalc Software, Ostend, Belgium). A value of p < 0.05 was considered statistically significant.

## Results

### Clinical characteristics of the study cohorts

The clinical characteristics of subjects were obtained from the electronic medical record system of the internal and external centers. The baseline data for the internal, external validation, and CMR validation datasets are detailed in Table 2.

**Table 2:** Study population of the internal cohort and external cohorts.

	Internal dataset			External dataset 1	External dataset 2	External dataset 3	External dataset 4	CMR validation dataset
	Development	Test	Total					
Number of patients	400	100	500	100	150	77	36	67
Number of videos	1200	300	1500	300	450	231	108	201
Demographics								
Age, median (IQR), y	52 (40, 61)	52 (44, 63)	52 (41, 62)	57 (47, 68)	57(47, 65)	52(26, 67)	47 (37, 59)	45 (34, 54)
Males, n (%)	293 (73.2)	78 (78.0)	371 (74.2)	74 (74.0)	101 (67.3)	47 (61.0)	19 (52.8)	55 (82.1)
BMI, median (IQR), kg/m^2^	23.2 (21.0, 25.5)	22.6 (20.5, 25.4)	23.1 (20.8, 25.5)	22.5 (21.0, 24.5)	25.7 (23.6, 28.1)	23.5 (21.3, 25.6)	23.7 (22.5, 26.1)	22.5 (19.8, 24.4)
Clinical diagnosis, n (%)								
Normal	96 (24.0)	15 (15.0)	111 (22.2)	29 (29.0)	70 (46.7)	43 (55.8)	21 (58.3)	/
Ischemic heart disease	103 (25.75)	23 (23.0)	126 (25.2)	39 (39.0)	80 (53.3)	34 (44.2)	15 (41.7)	9 (13.4)
Valvular heart disease	31 (7.75)	3 (3.0)	34 (6.8)	6 (6.0)	/	/	/	1 (1.5)
Cardiomyopathy	87 (21.75)	46 (46.0)	133 (26.6)	4 (4.0)	/	/	/	48 (71.6)
Congenital heart disease	11 (2.75)	1 (1.0)	12 (2.4)	1 (1.0)				
Hypertensive heart disease	19 (4.75)	5 (5.0)	24 (4.8)	5 (5.0)				
Other	53 (13.25)	7 (7.0)	60 (12.0)	16 (16.0)	/	/	/	9 (13.4)
Machine, n (%)								
EPIQ 7C	323 (80.75)	68 (68.0)	391 (78.2)	5 (5.0)	73 (48.7)	41 (53.2)	/	67 (100.0)
EPIQ 7	12 (3.0)	12 (12.0)	24 (4.8)	/	/	/	/	/
EPIQ 5C	/	/	/	/	2 (1.3)	/	/	/
EPIQ 5	59 (14.75)	13 (13.0)	72 (14.4)	/	/	25 (32.5)	/	/
EPIQ CVx	/	/	/	/	2 (1.3)	2 (2.6)	25 (69.4)	/
iE33	5 (1.25)	6 (6.0)	11 (2.2)	4 (4.0)	/	6 (7.8)	/	/
iE Elite	1 (0.25)	1 (1.0)	2 (0.4)	2 (2.0)	/	3 (3.9)	/	/
iU22	/	/	/	2 (2.0)	/	/	/	/
iU Elite	/	/	/	5 (5.0)	/	/	/	/
Vivid E95	/	/	/	64 (64.0)	1 (0.7)	/	11 (30.6)	/
Vivid E9	/	/	/	/	72 (48.0)	/	/	/
LISENDO 880	/	/	/	18 (18.0)	/	/	/	/

### Model performance

On the internal test dataset, the segmentation module of Echo-DFCNN was able to segment the left ventricle with high accuracy, with average DSC ranging from 0.92 to 0.95, and average ASD ranging from 1.71 mm to 1.97 mm (Table B in S1 Appendix).

In addition, on the internal test dataset, the optical flow module of Echo-DFCNN was able to trace the endocardial contour with high accuracy, with average HD ranging from 5.20 mm to 6.55 mm and average ASD ranging from 1.97 mm to 2.19 mm (Table C in S1 Appendix).

### Assessment of cardiac function

**Fig 4:**
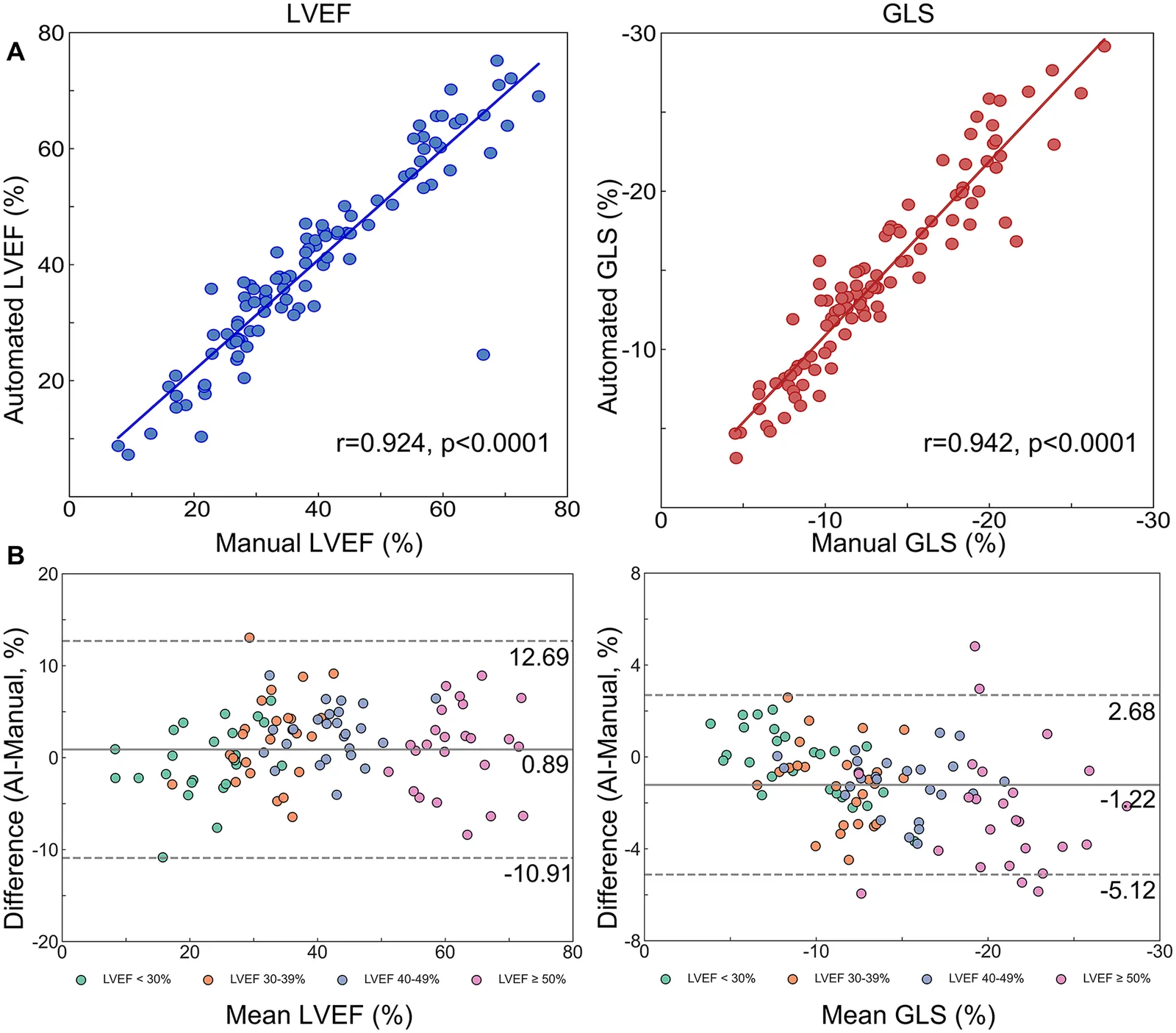
Correlation and agreement between the AI-predicted and manual measurements of LVEF and GLS. **A,** Correlation between the AI-predicted and manual measurements of LVEF and GLS on the internal test dataset (n = 100). **B,** Agreement between the AI-predicted and manual measurements of LVEF and GLS on the internal test dataset (n = 100), with data points stratified by LVEF ranges (LVEF < 30%, LVEF 30-39%, LVEF 40-49%, and LVEF ≥ 50%) to indicate different cardiac function statuses.

On the internal test dataset, the AI and manual measurements of LVEF demonstrated median values of 37.67% (IQR, 27.93 to 50.89) and 36.44% (IQR, 27.30 to 53.37), respectively (Table D in S1 Appendix). The median absolute error and mean absolute error of the Echo-DFCNN predictions of LVEF compared with manual measurements were 3.02% and 3.94%, respectively. The AI predictions were highly correlated with manual measurements (r = 0.924, p < 0.0001) (Fig 4 and Table 3). The AI predictions showed a high level of agreement compared to the manually measured values, with an ICC of 0.927 (95% CI, 0.894 to 0.950), with a bias of 0.89% and LOA of -10.91 to 12.69 (Fig 4 and Table 3). To further characterize the agreement for LVEF across different cardiac function ranges, we performed a stratified Bland-Altman analysis according to LVEF categories. The 95% LOA widths varied across different LVEF ranges: 15.30% for LVEF < 30%, 18.44% for LVEF 30–39%, 11.80% for LVEF 40–49%, and 38.20% for LVEF ≥ 50% (Table E in S1 Appendix). The widest LOA was observed in the LVEF ≥ 50% group, which was 3.24 times wider than that of the LVEF 40–49% group (Table E in S1 Appendix). Echo-DFCNN demonstrated median and mean absolute errors of 8.54% and 11.57% for LVEDV, and 7.21% and 10.48% for LVESV, respectively (Table 3). Echo-DFCNN achieved a sensitivity of 97.3% and a specificity of 96.2% when using a threshold of less than 50% to determine reduced left ventricular systolic function (Fig C in S1 Appendix).

**Table 3:** Correlation and agreement between the AI-predicted and manual LVEF and GLS on the internal test dataset (n = 100) and external validation datasets (n = 363).

	Absolute error (AI vs. Manual)		Correlation		Agreement		
	Median, (IQR)	Mean, (SD)	r	p-value	Bias	LOA	ICC, [95% CI]
Internal test dataset							
LVEDV, ml	8.54 (3.64, 16.54)	11.57 (10.46)	0.982	p < 0.0001	-1.36	[-31.89, 29.17]	0.984 [0.977, 0.989]
LVESV, ml	7.21 (3.81, 14.30)	10.48 (9.95)	0.976	p < 0.0001	-3.18	[-30.88, 24.52]	0.981 [0.972, 0.988]
LVEF, %	3.02 (1.54, 4.95)	3.94 (4.66)	0.924	p < 0.0001	0.89	[-10.91, 12.69]	0.927 [0.894, 0.950]
GLS, %	1.43 (0.66, 2.84)	1.83 (1.46)	0.942	p < 0.0001	-1.22	[-5.12, 2.68]	0.913 [0.791, 0.956]
External dataset 1							
LVEDV, ml	9.06 (3.73, 15.46)	11.23 (9.45)	0.936	p < 0.0001	3.43	[-24.62, 31.48]	0.956 [0.934, 0.971]
LVESV, ml	6.15 (2.29, 11.20)	8.03 (7.66)	0.916	p < 0.0001	2.22	[-19.14, 23.58]	0.969 [0.954, 0.979]
LVEF, %	2.80 (1.23, 5.37)	3.62 (3.02)	0.909	p < 0.0001	-0.16	[-9.41, 9.09]	0.941 [0.913, 0.960]
GLS, %	1.37 (0.50, 2.52)	1.84 (1.96)	0.892	p < 0.0001	-0.63	[-5.77, 4.51]	0.877 [0.819, 0.916]
External dataset 2							
LVEDV, ml	7.71 (4.58, 12.57)	9.83 (9.48)	0.825	p < 0.0001	-0.03	[-26.85, 26.78]	0.864 [0.816, 0.899]
LVESV, ml	4.39 (1.70, 8.96)	6.13 (6.05)	0.810	p < 0.0001	1.84	[-14.68, 18.35]	0.916 [0.885, 0.939]
LVEF, %	4.36 (2.07, 8.21)	5.84 (5.10)	0.665	p < 0.0001	-1.68	[-16.54, 13.18]	0.736 [0.650, 0.803]
GLS, %	1.80 (0.80, 3.46)	2.37 (2.09)	0.617	p < 0.0001	0.68	[-5.38, 6.74]	0.697 [0.601, 0.772]
External dataset 3							
LVEDV, ml	8.22 (3.01, 13.16)	10.58 (11.93)	0.939	p < 0.0001	2.64	[-28.26, 33.54]	0.940 [0.907, 0.961]
LVESV, ml	4.78 (2.53, 8.51)	7.24 (10.73)	0.935	p < 0.0001	1.53	[-23.70, 26.77]	0.952 [0.925, 0.969]
LVEF, %	4.08 (1.62, 6.67)	5.33 (5.66)	0.854	p < 0.0001	-0.61	[-15.85, 14.63]	0.868 [0.801, 0.914]
GLS, %	2.39 (0.78, 3.50)	2.66 (2.73)	0.736	p < 0.0001	-0.09	[-7.58, 7.41]	0.764 [0.652, 0.843]
External dataset 4							
LVEDV, ml	4.77 (2.45, 7.81)	5.93 (5.24)	0.975	p < 0.0001	3.10	[-11.27, 17.47]	0.954 [0.901, 0.977]
LVESV, ml	3.57 (1.63, 6.69)	4.00 (2.76)	0.948	p < 0.0001	1.06	[-8.32, 10.45]	0.945 [0.895, 0.972]
LVEF, %	2.39 (1.07, 5.11)	3.05 (2.39)	0.762	p < 0.0001	0.13	[-7.53, 7.79]	0.849 [0.724, 0.920]
GLS, %	1.63 (0.81, 2.36)	1.80 (1.29)	0.808	p < 0.0001	-0.51	[-4.77, 3.75]	0.789 [0.625, 0.886]

LVEDV, left ventricular end-diastolic volume; LVESV, left ventricular end-systolic volume; LVEF, left ventricular ejection fraction; GLS, global longitudinal strain; IQR, interquartile range; SD, standard deviation; r, Spearman correlation coefficient; CI, confidence interval; LOA, limits of agreement; ICC, intraclass correlation coefficients.

On the internal test dataset, the median value of GLS was -13.90% (IQR, -18.08 to -9.87) and -12.33% (IQR, -17.74 to -9.65) for the AI and manual measurements, respectively, with a median absolute error of 1.43% and a mean absolute error of 1.83% (Table 3). The two methods exhibited a high correlation (r = 0.942, p < 0.0001) and strong agreement (ICC = 0.913, 95% CI, 0.791 to 0.956; Bias = -1.22%, and LOA = -5.12% to 2.68%) (Fig 4 and Table 3). Stratified Bland-Altman analysis based on different cardiac function ranges showed that the 95% LOA widths for GLS were 5.85% in the LVEF < 30% group, 6.98% in the LVEF 30–39% group, 5.00% in the LVEF 40–49% group, and 10.44% in the LVEF ≥ 50% group (Table E in S1 Appendix). The LOA width in the LVEF ≥ 50% group was 2.09 times wider than that in the LVEF 40–49% group (Table E in S1 Appendix).

### Subgroup analysis

LVEF and GLS measured by AI and manual methods in different LVEF subgroups are shown in Table F in S1 Appendix. The Kruskal-Wallis H-test with Benjamini-Hochberg correction indicated no significant differences in LVEF measurement errors across different LVEF ranges (adjusted p = 0.432). Conversely, a significant overall difference was found for GLS measurement errors (adjusted p = 0.0002). Subsequent Mann-Whitney U tests with Benjamini-Hochberg correction revealed that the GLS measurement error was significantly higher in the LVEF ≥ 50% group compared to the LVEF < 30% group (adjusted p = 0.004), the LVEF 30–39% group (adjusted p = 0.049), and the LVEF 40–49% group (adjusted p = 0.004). The Spearman correlation and the ICC indicated that LVEF and GLS measured by AI and manual methods had high correlation and agreement in the LVEF < 30% and LVEF 40–49% groups, with moderate results observed in the LVEF 30–39% group (Table G in S1 Appendix). In contrast, the correlation and agreement of LVEF and GLS measured by the AI and manual methods were lower in the LVEF ≥ 50% group than in the other subgroups. Detailed results are shown in Table G in S1 Appendix.

**Fig 5:**
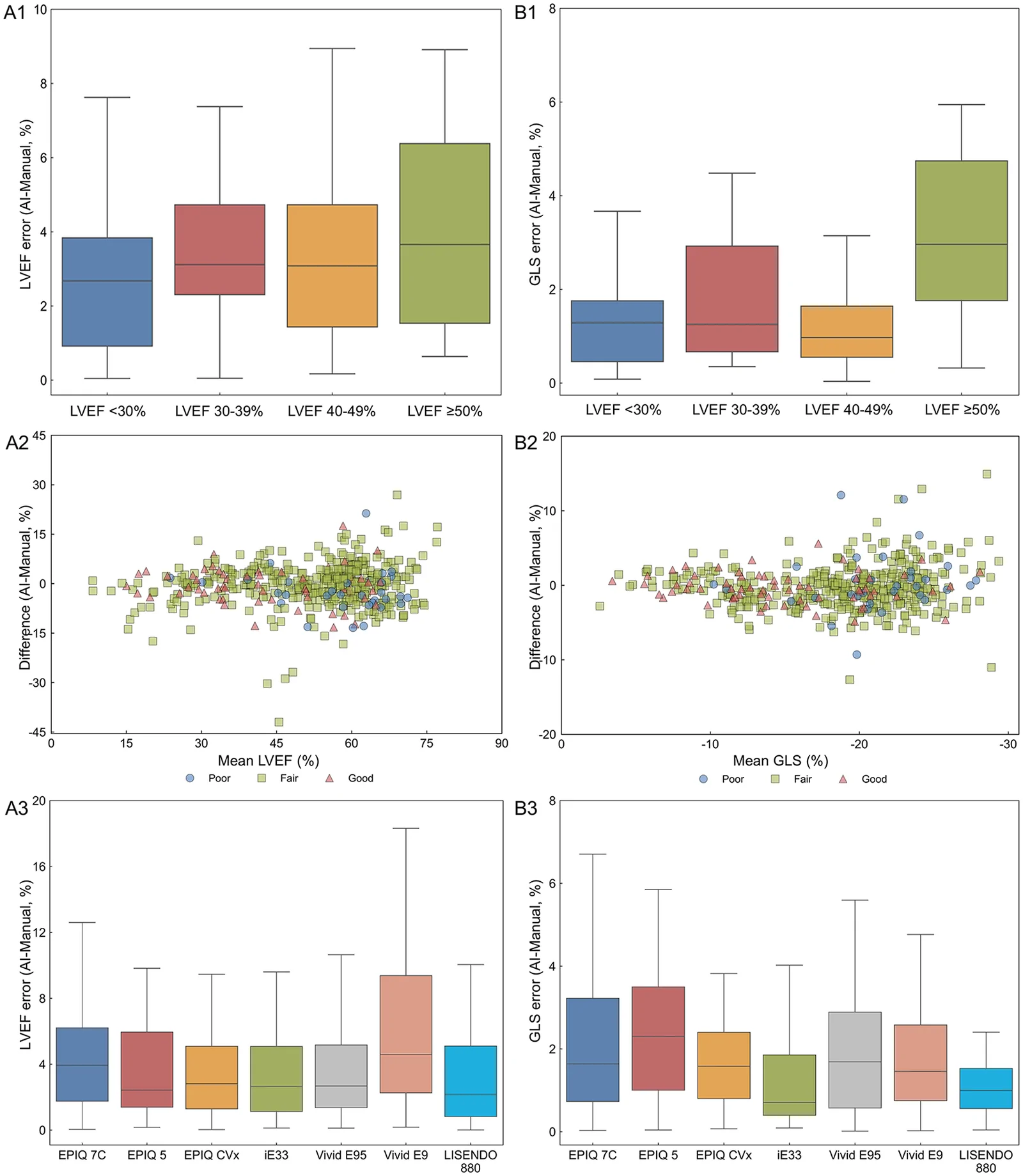
Subgroup analysis. **A1,** Errors between AI and manual measurements of LVEF in the subgroup of the test dataset (n = 100) by LVEF severity. **B1,** Errors between AI and manual measurements of GLS in the subgroup of the test dataset (n = 100) by LVEF severity. **A2,** Errors between AI and manual measurements of LVEF in the subgroup of the test dataset (n = 100) and external validation datasets (n = 363) by image quality. **B2,** Errors between AI and manual measurements of GLS in the subgroup of test dataset (n = 100) and external validation datasets (n = 363) by image quality. **A3,** Errors between AI and manual measurements of LVEF in the subgroup of the test dataset (n = 100) and external validation datasets (n = 363) by machine types. Machine types with sample sizes ≤15 were excluded from analysis. **B3,** Errors between AI and manual measurements of GLS in the subgroup of the test dataset (n = 100) and external validation datasets (n = 363) by machine types. Machine types with sample sizes ≤15 were excluded from analysis.

We categorized the internal test dataset and external validation datasets of 463 patients into three subgroups based on image quality (good, fair, and poor). The image quality evaluation method developed for LVEF and GLS measurements, demonstrated good inter-observer reliability, with ICCs of 0.873 (95% CI, 0.851 to 0.893) for LVEF and 0.888 (95% CI, 0.867 to 0.905) for GLS. Then, we evaluated the measurement errors of LVEF and GLS by AI and manual methods in different subgroups (Fig 5 and Tables H and I in S1 Appendix). Although a trend toward lower measurement errors with improved image quality was observed, the Kruskal-Wallis H-test with Benjamini-Hochberg correction revealed no significant difference in the overall measurement errors of LVEF and GLS across different image quality groups (LVEF: adjusted p = 0.837; GLS: adjusted p = 0.182). The Spearman correlation and the ICC revealed a high correlation and strong agreement between AI and manual measurements for LVEF and GLS in the good-quality group, with lower metrics in the fair- and poor-quality group (Tables H and I in S1 Appendix). This finding indicated that AI was able to analyze echocardiograms across diverse image qualities, but its reliability tended to decrease as image quality degraded.

To validate these findings, we performed a bidirectional sensitivity analysis by shifting the primary cut-offs by ±1 point. Across both alternative threshold sets, the trends in measurement errors, Spearman correlation coefficients, and ICCs for LVEF and GLS remained consistent with the primary analysis (Tables J–M in S1 Appendix). The further Kruskal-Wallis tests with Benjamini-Hochberg correction for both LVEF (adjusted p = 0.138 and adjusted p = 0.705) and GLS (adjusted p = 0.341 and adjusted p = 0.091) remained non-significant across both alternative threshold sets, reinforcing the findings of the primary analysis.

We proceeded to subgroup the internal test dataset and external validation datasets based on machine types (excluding machine types with a sample size ≤ 15), aiming to evaluate the performance of AI in measuring cardiac function across echocardiograms acquired by different ultrasound machines (Fig 5 and Table N in S1 Appendix). In terms of LVEF measurements, the Kruskal-Wallis H-test with Benjamini-Hochberg correction indicated a significant overall difference among machine types (adjusted p = 0.046). Mann-Whitney U tests with Benjamini-Hochberg FDR correction revealed that the LVEF measurement error was significantly higher in the Vivid E9 group compared to the Vivid E95 group (adjusted p = 0.010). The Kruskal-Wallis H-test for GLS measurement errors among different machine types was not statistically significant (adjusted p = 0.138), indicating robustness of Echo-DFCNN in measuring GLS across different ultrasound machines.

### Agreement between AI and commercial software QLAB

Comparison of AI with QLAB on the internal test dataset (n = 100) and external validation datasets (n = 363) showed that AI had strong correlation and acceptable agreement with QLAB for assessment of LVEF and GLS (LVEF: r = 0.850, ICC = 0.858, bias = -2.60%, LOA = -17.04% to 11.84%; GLS: r = 0.814; ICC = 0.831, bias = 0.50%, LOA = -6.14% to 7.14%). Detailed results are shown in Fig D and Table O in S1 Appendix.

We compared the time of assessing cardiac function parameters between AI and QLAB (Table P in S1 Appendix). The results showed that AI enabled simultaneous acquisition of LVEF and GLS in a median of 11.57 seconds (IQR, 10.87 to 12.41), which was faster than QLAB. QLAB required a median of 411.11 seconds (IQR, 362.53 to 471.06), comprising user interaction time of 394.41 seconds (IQR, 345.91 to 455.95) and software computation time of 15.76 seconds (IQR, 14.44 to 17.10). This time difference may be attributed to QLAB measuring LVEF and GLS separately by two independent functions. Therefore, the left ventricular endocardial contouring process needed to be repeated twice for the apical 2-chamber and 4-chamber views, respectively, which was more complex and time-consuming.

### Intra- and interobserver variability and agreement

On the subset of the internal test dataset (n = 50), Bland-Altman analysis demonstrated the variability of LVEF and GLS measured manually. Notably, AI performed the task autonomously without human input and thus had uniquely determined outputs when re-analyzing the same input. The intra-observer and inter-observer agreement for LVEF and GLS were good, as evidenced by high ICC, low bias, and narrow LOA. Detailed results are shown in Fig E and Table Q in S1 Appendix.

### Generalization to external medical centers

The generalization of Echo-DFCNN was further validated on four external datasets. The segmentation module of Echo-DFCNN was able to segment the left ventricle with an average DSC ranging from 0.79 to 0.91 and an average ASD ranging from 1.34 mm to 2.79 mm (Table B in S1 Appendix). In addition, the optical flow module of Echo-DFCNN was able to trace the endocardial contour with an average HD ranging from 5.34 mm to 9.24 mm and an average ASD ranging from 1.71 mm to 2.83 mm (Table C in S1 Appendix).

On the external datasets, the AI model demonstrated robust performance. For LVEF assessment, the median and mean absolute errors ranged from 2.39% to 4.36% and 3.05% to 5.84%, respectively, with strong correlation (r = 0.665 to 0.909, all p < 0.0001) and agreement (ICC = 0.736 to 0.941) compared to manual measurements (Table 3). Similarly, for GLS assessment, the median and mean absolute errors were 1.37% to 2.39% and 1.80% to 2.66%, respectively, also showing high correlation (r = 0.617 to 0.892, all p < 0.0001) and agreement (ICC = 0.697 to 0.877) with manually measured values (Table 3).

### Cross-modal validation of Echo-DFCNN using CMR

For the CMR validation dataset (n = 67), clinical stability between echocardiography and CMR examinations was assessed. As detailed in Table R in S1 Appendix, there were no acute clinical events, such as cardiac surgery, percutaneous coronary intervention, cardiac implantable electronic device implantation, catheter ablation, acute decompensated heart failure, or acute coronary syndrome, during the interval between the two imaging modalities. Furthermore, no patients required admission to the hospital between scans, and 83.6% (56/67) of the patients were inpatients for both examinations, remaining under continuous clinical monitoring.

In the CMR validation dataset, LVEF and GLS were measured using three methods: AI, QLAB, and CMR. Results showed that both the AI model and QLAB analysis overestimated LVEF and GLS compared with CMR. Detailed results are shown in Table S in S1 Appendix.

With regard to LVEF measurement, the median absolute error and mean absolute error between AI and CMR measurements were 4.89% and 5.09%, and the median absolute error and mean absolute error between QLAB and CMR measurements were 5.61% and 6.23%, respectively (Table 4). For GLS measurement, the median absolute error and mean absolute error between AI and CMR measurements were 1.64% and 1.69%, and the median absolute error and mean absolute error between QLAB and CMR measurements were 1.37% and 1.51%, respectively (Table 4).

**Table 4:** Correlation and agreement of LVEF and GLS between different techniques on the CMR validation dataset (n = 67).

	Absolute error		Correlation		Agreement		
	Median, (IQR)	Mean, (SD)	r	p-value	Bias	LOA	ICC, [95% CI]
AI vs. CMR							
LVEF, %	4.89 (2.22, 7.81)	5.09 (3.26)	0.775	p < 0.0001	2.31	[-8.70, 13.32]	0.909 [0.835, 0.947]
GLS, %	1.64 (0.73, 2.55)	1.69 (1.02)	0.740	p < 0.0001	-0.58	[-4.30, 3.14]	0.880 [0.806, 0.927]
QLAB vs. CMR							
LVEF, %	5.61 (3.17, 9.30)	6.23 (3.70)	0.771	p < 0.0001	4.81	[-5.87, 15.49]	0.860 [0.469, 0.944]
GLS, %	1.37 (0.70, 2.21)	1.51 (1.02)	0.728	p < 0.0001	-0.51	[-3.96, 2.94]	0.885 [0.815, 0.929]

LVEF, left ventricular ejection fraction; GLS, global longitudinal strain; IQR, interquartile range; SD, standard deviation; r, Spearman correlation coefficient; CI, confidence interval; LOA, limits of agreement; ICC, intraclass correlation coefficients.

For LVEF measurement, the AI model demonstrated a good correlation with CMR analysis (r = 0.775, p < 0.0001). Similarly, QLAB derived LVEF correlated well with CMR values (r = 0.771, p < 0.0001). The performance of the AI model for measuring LVEF is comparable to QLAB’s method (r = 0.775 vs.0.771, p = 0.955). For GLS measurement, the AI model (r = 0.740, p < 0.0001) and QLAB (r = 0.728, p < 0.0001) also demonstrated good correlations with CMR measurements. The AI model had similar performance to the QLAB method for GLS evaluation (r = 0.740 vs. 0.728, p = 0.883). Detailed results are shown in Table 4. Bland-Altman analysis showed that the bias between the AI model and CMR for LVEF and GLS was relatively small (Table 4). The AI model also exhibited high ICC, indicating good agreement with CMR.

### Computational benchmarking of Echo-DFCNN

To characterize the computational requirements and deployment feasibility of Echo-DFCNN, we evaluated its performance in 25 patients randomly selected from the internal test and external datasets under different GPU- and CPU-based configurations (Table T in S1 Appendix). Using an NVIDIA GeForce RTX 3090 GPU with four helper CPU threads, the median inference time for all three required views was 14.41 seconds (IQR, 14.03 to 15.71) per patient. CPU-only inference was substantially slower, with median inference times of 1259.79 seconds (IQR, 1178.09 to 1403.50) using 32 cores, 1948.58 seconds (IQR, 1831.46 to 2169.39) using 16 cores, and 3383.87 seconds (IQR, 3207.57 to 3780.04) using 8 cores on an Intel Xeon Silver 4216 processor. All tested configurations successfully completed inference for all 25 patients.

These results suggest that Echo-DFCNN may be suitable for deployment as a centralized GPU-based post-processing service. Although CPU-only execution was technically feasible, its substantially longer processing time may limit its practical application in lower-resource settings without GPU access. Future work will focus on model lightweighting, computational optimization, and hardware-adaptive acceleration to improve inference efficiency across a broader range of resource settings.

### The ablation study

**Fig 6:**
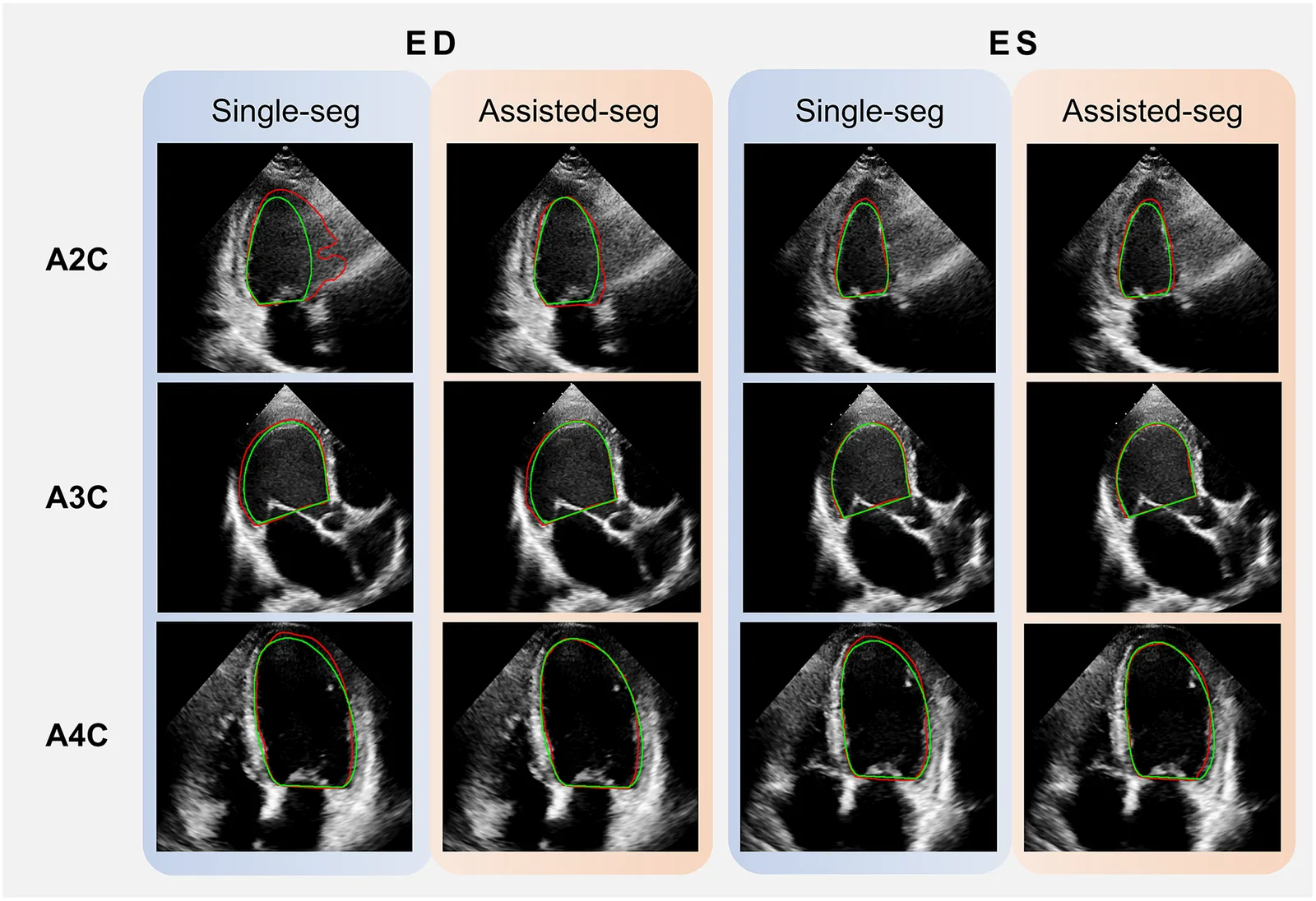
Visualization of the improvement in segmentation with the optical flow module in the apical 2-chamber, 3-chamber, and 4-chamber views. Single-seg, segmentation without optical flow network assistance; Assisted-seg, segmentation with optical flow network assistance; ED, end-diastole; ES, end-systole. Predicted results are shown in red, and manual annotations in green.

To evaluate the contribution of optical flow to Echo-DFCNN, we compared the segmentation with optical flow network assistance (Assisted-seg) with the segmentation without optical flow assistance (Single-seg). The visualization results of the enhancing effect are shown in Fig 6.

The LVEF measurement errors of the Assisted-seg and the Single-seg methods on the internal test dataset and the external validation datasets are shown in Table U in S1 Appendix. Further analysis was conducted on the internal test dataset (n = 100) and external validation datasets (n = 363) stratified by image quality (Table 5 and Fig F in S1 Appendix). Across all image quality categories, the Assisted-seg method demonstrated a trend of lower mean absolute error and median absolute error compared to the Single-seg method (Table 5). Specifically, the Assisted-seg method achieved a significantly lower error compared to the Single-seg method (adjusted p < 0.001) for fair-quality images (n = 373). However, no significant differences were observed between the two methods for good-quality images (n = 56, adjusted p = 0.144) and poor-quality images (n = 32, adjusted p = 0.144), which may be attributed to the limited sample sizes in the two groups (Table 5).

**Table 5:** Comparison of errors in LVEF measurements between Single-seg and Assisted-seg on the internal test dataset (n = 100) and external validation datasets (n = 363) by image quality.

Strategy	Absolute error (AI vs. Manual)	Good (n = 56)	Fair (n = 373)	Poor (n = 32)
Single-seg	Median, (IQR), %	4.09 (1.32, 6.82)	4.15 (2.37, 7.00)	4.79 (2.95, 7.22)
	Mean, (SD), %	4.94 (4.39)	5.51 (5.61)	5.47 (3.12)
Assisted-seg	Median, (IQR), %	2.90 (1.60, 5.28)	3.51 (1.41, 6.45)	3.63 (2.03, 6.04)
	Mean, (SD), %	4.23 (3.68)	4.69 (4.82)	4.85 (4.59)
p-value		Adjusted p = 0.144	Adjusted p < 0.001	Adjusted p = 0.144

Single-seg, segmentation without optical flow network assistance; Assisted-seg, segmentation with optical flow network assistance; IQR, interquartile range; SD, standard deviation. Note that one case from external dataset 2 (in the fair-quality group) and one case from external dataset 3 (in the poor-quality group) were excluded from the analysis due to Single-seg method failure.

Notably, two cases (one from external dataset 2 and one from external dataset 3) were excluded from the analysis due to Single-seg method failure. We conducted an individual analysis of these two cases. As shown in Fig G in S1 Appendix, the Single-seg method yielded an AI-predicted LVEDV smaller than the LVESV, resulting in a negative LVEF. In contrast, the Assisted-seg method demonstrated improved segmentation with the aid of optical flow.

In addition, Assisted-seg had a median model inference time of 3.93 seconds (IQR, 3.75 to 4.14) compared to Single-seg’s 2.10 seconds (IQR, 2.09 to 2.19) (Table V in S1 Appendix). This indicated that while the integration of optical flow extended the processing time, the resulting duration was still considered within an acceptable range for practical applications.

## Discussion

This study developed a dual-flow AI model based on segmentation and optical flow algorithms called Echo-DFCNN. The integrated pipeline of the segmentation and optical flow modules enables simultaneous measurements of LVEF and GLS. Indeed, the strategy of collaboratively processing complementary information via dual-flow architectures has been widely validated across various domains, including medical imaging [40] and natural scene analysis [41]. The model requires apical 2-chamber, apical 3-chamber, and apical 4-chamber echocardiographic videos as input, achieving results in approximately 12 seconds. Echo-DFCNN enables automated analysis of echocardiographic videos and measurement of cardiac function without the necessity for manual delineation of the endocardium, which is time-consuming. Echo-DFCNN has a uniquely determined output for the same input image, eliminating inter-observer and intra-observer variability. The performance of Echo-DFCNN was validated across 4 geographically distinct cohorts. More importantly, it underwent cross-modal validation using CMR imaging, as a gold standard for left ventricular function assessment, and shows good correlation and high agreement with CMR measurements. In addition, Echo-DFCNN demonstrated its potential for robust performance across a wide range of cardiac function, different image qualities, and machine types in the study cohorts. These findings support the potential use of Echo-DFCNN for cardiac function evaluation in clinical applications.

In this study, optical flow through intermediate frames provided the segmentation algorithm with continuous myocardial motion information, aiming to improve the consistency of left ventricular segmentation results. To validate the effectiveness of the optical flow assistance, we performed comparative analyses stratified by image quality. Across all image quality categories (good, fair, and poor), the Assisted-seg method demonstrated consistently lower mean and median absolute errors in LVEF measurements compared to the Single-seg method. Statistical significance was reached specifically in fair-quality images (adjusted p < 0.001), whereas no significant differences were observed in good- and poor-quality images, which may be attributed to the limited sample sizes in these groups. The method leverages optical flow to extract additional temporal information from subsequent frames, which demonstrated significant clinical benefit in fair-quality images, while its role in good- and poor-quality images requires further exploration with larger cohorts. At the same time, the segmentation aids optical flow by providing an initial endocardial contour, thereby automatically measuring GLS.

Based on the calculation approach, previous studies on LVEF assessment can be primarily divided into two categories: the single-plane Simpson’s method relying on the A4C view, and the biplane Simpson’s method utilizing both A2C and A4C views (Table W in S1 Appendix). Ouyang et al. [17] and Dai W et al. [18] both adopted the single-plane strategy, achieving mean absolute errors of 4.1% and 4.17%, respectively, on the EchoNet-Dynamic dataset. However, the single-plane Simpson’s method assumes that the left ventricular geometry observed in the A4C view is representative of the entire ventricle, neglecting shape variations in the orthogonal plane. In contrast, the biplane Simpson’s method recommended by clinical guidelines [6] utilizes two orthogonal apical views (A2C and A4C) to more comprehensively capture left ventricular geometry and reduce errors arising from single-plane geometric assumptions. Among studies using the biplane Simpson’s method, existing approaches have predominantly relied on frame-by-frame spatial segmentation networks, with their primary contributions centered on cross-cohort clinical validation [19,20,42].

Echo-DFCNN exhibited high correlation and agreement with manual measurements (r = 0.924, ICC = 0.927, mean absolute error of 3.94%, median absolute error of 3.02%) on the internal test dataset. Across the four external centers, the median and mean absolute errors ranged from 2.39% to 4.36% and 3.05% to 5.84%, respectively. Echo-DFCNN followed clinical guidelines by adopting the biplane Simpson’s method, enabling a clinically standardized evaluation of cardiac function. In terms of the model architecture, Echo-DFCNN integrated optical flow algorithms with the segmentation network, leveraging inter-frame motion information to improve segmentation accuracy and consistency. This provides an advantage compared to independent segmentation approach such as U-Net.

For GLS assessment, the most commonly used method is the traditional speckle tracking approach, which tracks bright speckle patterns frame-by-frame to analyze myocardial motion. However, this method has limitations: it is sensitive to occlusions or noise, and its performance significantly drops when speckle signals weaken or are lost. Additionally, its reliance on frame rate makes it challenging to accurately track at low frame rates or analyze motion effectively. The EACVI/ASE/Industry Task Force to Standardize Deformation Imaging studied variations in GLS measurements among vendors using traditional speckle tracking methods [15,16]. By comparison, optical flow tracking methods offer advantages. Rather than relying exclusively on bright speckles, this method identifies and tracks dense pixel points, generating a more comprehensive and accurate representation of motion. Optical flow algorithms learn context-specific matching rules and analyze continuous inter-frame motion, enabling reliable myocardial tracking even in low-quality echocardiographic images (e.g., noisy or low-contrast images). Importantly, these methods are independent of frame rate, enhancing their flexibility and robustness in diverse imaging scenarios.

Several studies have explored the use of optical flow algorithms for GLS assessment (Table W in S1 Appendix). Salte IM. et al. [21] proposed an AI model based on an optical flow algorithm using echocardiogram videos of 200 patients, achieving a median absolute error of 1.4% and a mean absolute error of 1.8% for GLS measurement. They further integrated this algorithm into a deep learning platform and conducted a prospective study involving 50 patients [22]. The AI measurements and manual measurements exhibited a high correlation (r = 0.94) and agreement (Bias = -1.3%, and LOA = -3.5% to 0.8%). Evain E. et al. [23] presented an AI model on a synthetic virtual dataset and tested it on 30 patients. The mean absolute error of automated GLS measurements was 2.5%. While these studies demonstrated the potential of optical flow algorithms for automatic GLS measurements, the methods still face limitations in model generalizability and performance in complex myocardial motion scenarios. For instance, the studies by Salte IM. et al. [21,22] were based on a dataset of a relatively small sample size that may limit the model’s adaptability to larger and more diverse populations. Similarly, while Evain E. et al. [23] tested their approach on synthetic virtual datasets, their clinical validation involved only 30 patients and did not adequately account for variations in image quality in real-world scenarios. In contrast, our study conducted a comprehensive validation of Echo-DFCNN, testing its performance not only across four external cohorts and various subgroups (including a wide range of cardiac functions, image quality, and machine types) but also through cross-modal validation against CMR imaging, the gold standard for left ventricular function assessment, further confirming its accuracy and robustness. Our study revealed that Echo-DFCNN showed acceptable GLS measurement errors (median absolute error 1.43%, mean absolute error 1.83%) on the internal test dataset, slightly surpassing previous studies and demonstrating its high accuracy in tracking endocardial contours. These findings further confirmed the model’s potential in automated analysis of echocardiography and measurement of cardiac functional parameters.

This study conducted subgroup analyses based on different ranges of LVEF, image quality, and ultrasound machine types to further explore the AI model’s performance. Firstly, LVEF measurement errors remained statistically stable across different LVEF ranges, although stratified Bland-Altman analysis revealed slightly wider limits of agreement in the LVEF ≥ 50% group. In contrast, GLS measurements in the LVEF ≥ 50% group demonstrated both significantly increased measurement errors and notably reduced agreement, warranting cautious clinical interpretation. Especially in the group with LVEF ≥ 50%, there were 5 cases (20%) of patients with hypertrophic cardiomyopathy. In these cases, hypertrophic myocardium, papillary muscle thickening, and increased trabeculae carneae obscured endocardial visualization, while systolic compression of cardiac structures further challenged endocardial identification, impacting measurement accuracy.

Secondly, Echo-DFCNN showed its capability to analyze echocardiograms across diverse image qualities, but its reliability tended to decrease as image quality degraded. This trend was further supported by our bidirectional sensitivity analysis. However, the relatively small sample sizes for the good- and poor-quality groups limited the precision of subgroup-specific performance estimates and the statistical power of between-group comparisons, necessitating cautious interpretation of these findings.

Finally, when categorized by machine types, significant differences in LVEF measurement errors were observed among the Vivid E9 and the Vivid E95 groups. In contrast, GLS measurements by AI remained stable across different ultrasound machines, which indicates promising cross-platform generalizability. Notably, the findings related to machine types with smaller sample sizes (e.g., EPIQ 5, EPIQ CVx, and LISENDO 880) should be interpreted with caution. Given the predominant inclusion of Philips systems in this study’s machine types, broader generalizability to other manufacturers’ equipment necessitates further validation with larger datasets. In general, this study provides meaningful insights into the reliability of Echo-DFCNN in clinical applications.

Our study demonstrated that Echo-DFCNN performed comparably to QLAB in measuring LVEF and GLS, showing a high correlation with CMR. Additionally, Bland-Altman analysis revealed small biases between the AI model and CMR analysis, further confirming its stability. These findings were consistent with previous studies, such as those by Sveric KM et al. [43] and Samtani R et al. [44], which showed that AI methods exhibited a high correlation with CMR in LVEF measurement and outperformed post-processing software manual methods. However, the AI used in those studies was commercial automated analysis software rather than self-developed models. In addition, to the best of our knowledge, no previous studies have validated self-developed AI models for automatic GLS measurement using a CMR cohort.

However, the results also indicated that both the AI model and QLAB analysis tended to overestimate LVEF and GLS when compared with CMR. This could be attributed to 43 (64.2%) of the patients in the CMR cohort presenting with dilated cardiomyopathy. In such cases, the left ventricle might extend beyond the echocardiographic sector imaging area. Both AI and QLAB might rely on the visible segments to make inferences, potentially leading to inaccurate endocardial tracing.

### Study limitations

There are some limitations in this study. First, in the evaluation of inter-observer variability, two observers measured the same video, which neglected the variability in image acquisition in clinical practice and led to the underestimation of inter-observer variability. Second, while the development and test cohorts included patients with specific clinical characteristics that affect image quality and a variety of clinical diagnoses, the limited sample sizes for these populations may imply potential insufficient training and testing of the model on these conditions. Third, the machine types in this study were predominantly Philips ultrasound systems, limiting the generalizability to other machine types. The assessment of Echo-DFCNN’s performance on non-Philips ultrasound systems should be considered preliminary, and substantially larger prospective validation studies with balanced representation across manufacturers are essential before clinical deployment on diverse ultrasound devices. Fourth, the model exhibited variable reliability across cardiac function ranges, characterized by reduced agreement for LVEF and GLS in the LVEF ≥ 50% group. Future studies with larger sample sizes are warranted to optimize performance across various ranges. Fifth, the limited sample size in the subgroup analysis restricts the statistical power for evaluating the measurement agreement of Echo-DFCNN in different LVEF ranges, image qualities, and machine types. Future research should enlarge the sample size to evaluate the model’s performance in various groups comprehensively. Sixth, although the CMR validation dataset demonstrated clinical stability between echocardiography and CMR examinations with no acute events, routine adjustments in cardiovascular medications during this interval could still potentially introduce confounding. Finally, the AI model’s performance in patients with dilated cardiomyopathy may be limited due to the left ventricle extending beyond the echocardiographic sector imaging area. Future studies should incorporate more patient data with complex left ventricular morphology to optimize model performance in these challenging cases.

## Conclusions

This study proposes a dual-flow AI model based on segmentation and optical flow algorithms called Echo-DFCNN for automatic analysis of echocardiogram videos. Echo-DFCNN achieves simultaneous and precise assessment of LVEF and GLS in the study cohorts, demonstrating its potential for robust performance across different cardiac functions, image qualities and machine types. The application of this model has the potential to improve the efficiency of radiologists and provide support for more effective management of patients with cardiovascular diseases.

## Supporting information

S1 Appendix Supplemental methods, results, discussion, figures, tables, and references. (DOCX)

## Abbreviations

LVEF: left ventricular ejection fraction
LVEDV: left ventricular end-diastolic volume
LVESV: left ventricular end-systolic volume
GLS: global longitudinal strain
AI: artificial intelligence
ED: end-diastolic
ES: end-systolic
CMR: cardiac magnetic resonance
DSC: Dice similarity coefficient
ASD: average surface distance
HD: Hausdorff distance
ICC: intraclass correlation coefficients
LOA: limits of agreement
SD: standard deviation
IQR: interquartile range
AUC: area under the curve
