# Supplementary material for: Dual-flow convolutional neural network for automatic measurement of left ventricular ejection fraction and global longitudinal strain in echocardiography
Source: PLOS Digit Health. 2026 Aug 28;5(8):e0001128. doi: 10.1371/journal.pdig.0001128 (PMC13524239; doi:10.1371/journal.pdig.0001128)
Supplement: Supplementary file 1 [file pdig.0001128.s001.docx]

S1 Appendix

1. SUPPLEMENTAL METHODS	2

1.1 Datasets	2

1.1.1 Echocardiogram acquisition	2

1.1.2 CMR image acquisition	2

1.1.3 Baseline information collection	2

1.2 Preprocessing	4

1.3 Annotation	5

1.4 Dataset splitting strategy	6

1.5 Dual-flow convolutional neural network	6

1.5.1 The optical flow module	7

1.5.2 The segmentation module	9

1.5.3 Loss function	11

1.5.4 Evaluation of model performance	12

1.6 PRIME checklist	14

2. SUPPLEMENTAL RESULTS	16

2.1 Model performance	16

2.2 Assessment of cardiac function	17

2.3 Agreement categorized using LVEF, image quality, and machine types	18

2.4 In between methods agreement AI vs. commercial software QLAB	23

2.5 Intra- and interobserver variability and agreement	25

2.6 Cross-modal validation of Echo-DFCNN using CMR	26

2.7 Computational benchmarking of Echo-DFCNN	27

2.8 The ablation study	27

3. SUPPLEMENTAL DISCUSSION	31

4. SUPPLEMENTAL REFERENCES	33

1. SUPPLEMENTAL METHODS

1.1 Datasets

1.1.1 Echocardiogram acquisition

In the internal dataset, the echocardiograms were recorded using commercial ultrasound systems (EPIQ 5, EPIQ 7, EPIQ 7C, iE33, or iE Elite systems, Philips). Ultrasonic probes were S5_1, S8_3, and X5_1, with the frame rate set at 48 ± 5 frames per second.

In the external dataset, more different types of ultrasound acquisition systems (LISENDO 880 system, FUJIFILM; EPIQ 5, EPIQ 5C, EPIQ 7C, EPIQ CVx, iE33, iE Elite, iU Elite or iU22 systems, Philips; Vivid E9 or Vivid E95 systems, GE Vingmed Ultrasound) were included. Ultrasonic probes consisted of S5_1, X5_1, and S121. The frame rates for each external dataset were as follows: 1) External dataset 1: 50±2 frames per second; 2) External dataset 2: 53±1 frames per second; 3) External dataset 3: 51±5 frames per second; 4) External dataset 4: 53±5 frames per second.

In the CMR validation dataset, the echocardiograms were recorded using the commercial ultrasound system (EPIQ 7C, Philips). Ultrasonic probes were S5_1, S8_3, and X5_1, with the frame rate set at 46 ± 4 frames per second.

All echocardiographic examinations were conducted in accordance with American Society of Echocardiography (ASE) guidelines[1, 2].

1.1.2 CMR image acquisition

In the CMR validation dataset, CMR images were acquired using a 3.0T MRI scanner (MAGNETOM, Siemens Healthineers, Erlangen, Germany). Breath-holding training was conducted before the scans. In a supine position during a resting state, balanced steady-state free precession sequences were used to acquire dynamic cine images of the left ventricle in short-axis views (from apex to base) and long-axis views (four-chamber, two-chamber, and three-chamber).

CMR scan parameters: 1) Short-axis: repetition time 46.40 ms, echo time 1.21 ms, slice thickness 8 mm, field of view 298 × 340 mm², matrix 224 × 256, flip angle 80°. 2) Long-axis: repetition time 38.09 ms, echo time 1.21 ms, slice thickness 6 mm, field of view 340 × 340 mm², matrix 256 × 256, flip angle 47°.

1.1.3 Baseline information collection

Demographic characteristics (gender, age, height, weight) and clinical data (disease category) were obtained from the electronic medical record system of the internal and external medical centers. All the diagnoses were clinically confirmed. The clinical diagnoses represent the primary clinical diagnosis for the current admission or outpatient examination. We exported echocardiogram videos of patients from the Picture Archiving and Communication System, stored in DICOM format.

In the internal dataset, a total of 500 patients included 7 disease categories: 1) Healthy volunteers (n=111; no significant cardiac disease); 2) Patients with ischemic heart disease (n=126), including cases assessed at various time points since the onset of current illness: within 7 days (37 cases), 7 days to 1 month (14 cases), 1 month to 1 year (30 cases), and over 1 year (45 cases); 3) Patients with valvular heart disease (n=34), including aortic valve disease (7 cases), aortic valve replacement (3 cases), mitral valve disease (11 cases), tricuspid valve disease (5 cases), and multi-valve disease (8 cases); 4) Patients with cardiomyopathy (n=133), including dilated cardiomyopathy (97 cases), hypertrophic cardiomyopathy (15 cases), restrictive cardiomyopathy (1 case), left ventricular noncompaction (19 cases), and cardiac amyloidosis (1 case); 5) Congenital heart disease (n=12); 6) Hypertensive heart disease (n=24); 7) Others (n=60, excluding the six types mentioned above, they were classified as others, including : ① Other cardiovascular diseases (n=9): Arrhythmias (6 cases), aortic dissection (1 case), myocarditis (1 case), and pulmonary embolism (1 case); ② Systemic diseases with potential cardiovascular involvement (n=38): Renal insufficiency (5 cases), diabetes mellitus (3 cases), diabetes mellitus with renal insufficiency (2 cases), rheumatic and autoimmune diseases (6 cases), malignant tumors (18 cases, including hematological malignancies 7 cases, pulmonary malignancies 4 cases, endocrine malignancies 2 cases, gastrointestinal malignancies 2 cases, reproductive tract malignancies 1 case, thymoma 1 case, and cutaneous malignancies 1 case), sepsis (1 case), respiratory failure (1 case), dyslipidemia (1 case), and nodular goiter (1 case); ③ Other non-cardiac and non-systemic diseases (n=13): Vocal fold polyps (1 case), oral squamous papilloma (1 case), retinal detachment (1 case), epistaxis (1 case), gastric ulcer with perforation (1 case), gallbladder adenomyomatosis (1 case), fatty liver (1 case), cervical spondylotic myelopathy (1 case), lumbar spinal stenosis (1 case), lumbar disc herniation (1 case), cerebral stroke (1 case), intracranial aneurysm (1 case), and cerebral dysfunction (1 case).

The external dataset 1 included 100 cases: 1) Healthy volunteers (n=29); 2) Patients with ischemic heart disease (n=39), including cases assessed at various time points since the onset of current illness: within 7 days (13 cases), 7 days to 1 month (4 cases), 1 month to 1 year (8 cases), and over 1 year (14 cases); 3) Patients with valvular heart disease (n=6), including aortic valve disease (3 cases), aortic valve replacement (2 cases),and mitral valve replacement (1 case); 4) Patients with cardiomyopathy (n=4), including dilated cardiomyopathy (2 cases) and cardiac amyloidosis (2 cases); 5) Congenital heart disease (n=1); 6) Hypertensive heart disease (n=5); 7) Others (n=16), including: ① Other cardiovascular diseases (n=1): Arrhythmias (1 case), ② Systemic diseases with potential cardiovascular involvement (n=12): Renal insufficiency (5 cases), renal insufficiency combined with hypertensive heart disease (4 cases), viral pneumonia (2 cases), diabetes mellitus combined with hypertensive heart disease (1 case); ③ Other non-cardiac and non-systemic diseases (n=3): Benign prostatic hyperplasia (1 case), transient ischemic attack (1 case), lumbar disc herniation (1 case).

The external dataset 2 included 150 cases: 1) Healthy volunteers (n=70); 2) patients with ischemic heart disease (n=80), including cases assessed at various time points since the onset of current illness: within 7 days (13 cases), 7 days to 1 month (44 cases), 1 month to 1 year (21 cases), and over 1 year (2 cases).

The external dataset 3 included 77 cases: 1) Healthy volunteers (n=43); 2) Patients with ischemic heart disease (n=34), including cases assessed at various time points since the onset of current illness: within 7 days (13 cases), 7 days to 1 month (16 cases), 1 month to 1 year (4 cases), and over 1 year (1 case). The external dataset 4 included 36 cases: 1) Healthy volunteers (n=21); 2) Patients with ischemic heart disease (n=15), including cases assessed at various time points since the onset of current illness: within 7 days (1 case), 7 days to 1 month (4 cases), 1 month to 1 year (7 cases, and over 1 year 3 cases).

The CMR validation dataset included 67 cases: 9 cases with ischemic heart disease, 1 with aortic valve disease, 3 with hypertrophic cardiomyopathy, 43 with dilated cardiomyopathy, 1 with restrictive cardiomyopathy, 1 with arrhythmogenic right ventricular cardiomyopathy, and 9 with others (1 with myocarditis and 8 with post-heart transplantation).

1.2 Preprocessing

Data preprocessing consisted of two steps: 1) anonymization; 2) extraction of the region of interest (ROI) in echocardiogram videos.

Step 1: Anonymization of echocardiographic videos acquired for different ultrasound devices. The images were processed by the OpenCV library using dilation and erosion operations to remove patients' sensitive information (e.g., name, etc.) from the DICOM tag files.

Step 2: Extraction of ROI in echocardiography. The specific procedure was to extract the ROI from the single-sector echocardiographic videos captured by various ultrasound devices (including EPIQ 5, EPIQ 7, EPIQ 7C, IE Elite, iE33, etc.) based on the prior mask images. The images were then cropped to enhance the ratio of the ROI to the background, which aids the AI algorithm in quantitative echocardiogram analysis.

1.3 Annotation

The annotation team consisted of two radiologists with 3-10 years of experience and one senior radiologist with over 10 years of experience. Firstly, the internal and external datasets were independently annotated by one radiologist (Observer 1). Secondly, observer variability was assessed on a subset of the internal test dataset (n=50). For intra-observer variability, the same observer (Observer 1) reanalyzed the data one month after the initial analysis, ignoring the previous measurement results. For inter-observer variability, two observers (Observer 1 and Observer 2) independently completed the annotation. All annotation results underwent quality control by the senior radiologist. All videos were annotated using ITK-SNAP (version 3.8.0). During annotation, the radiologist selected a representative cardiac cycle in each video and manually tracked the left ventricular endocardium at ED and ES.

In the CMR validation dataset, the QLAB measurements were manually performed by a senior radiologist with over 10 years of experience, while the CMR measurements were manually performed by a senior radiologist with over 10 years of experience. The CMR measurements were conducted using the CVI42 workstation (CVI42, Circle, Calgary, Canada, version 5.1.1).

According to the recommendations of the American Society for Echocardiography, the papillary muscles and trabeculae were included in the left ventricular cavity^1^. ED is defined as the frame with the largest left ventricular volume, and ES is defined as the frame with the smallest left ventricular volume^1^.

Considering the different types of ultrasound devices, all the echocardiographic videos and labels were resized to 512×512 pixels through linear interpolation, which was beneficial for subsequent deep-learning processing.

1.4 Dataset splitting strategy

The internal dataset was randomly stratified into the development dataset (n=400) and the test dataset (n=100) in an 8:2 ratio based on LVEF. The development dataset was then randomly divided into training (n=280) and validation (n=120) in a 7:3 ratio.

On the internal test dataset, we compared the LVEF and GLS measured by Echo-DFCNN with manual measurements to evaluate the model performance. We also performed a subgroup analysis on the internal test dataset to assess the performance of the AI model across various cardiac functions.

We divided a random subset of 50 patients in the test dataset for inter-observer and intra-observer variability analysis.

The external validation datasets included data from four centers, which included 363 patients containing 1,089 videos. We performed subgroup analysis on the internal test dataset and external validation datasets to assess the performance of the AI model across different image quality and machine types. We further compared the AI measurements with QLAB Advanced Quantification Software (version 13.0, Philips) on the internal test dataset and external validation datasets.

The CMR validation dataset included 201 videos from 67 patients from the internal center. AI-predicted values, QLAB measurements, and CMR measurements were collected, with CMR measurements serving as the gold standard for cardiac function assessment to evaluate the performance of the AI model.

It was worth mentioning that the dataset included in this study did not include multiple examinations of the same patient, so there was no risk of data leakage.

1.5 Dual-flow convolutional neural network

This study developed a dual-flow neural network based on the segmentation algorithm and the optical flow algorithm called Echo-DFCNN. Echo-DFCNN consisted of two modules. The first module was based on the RAFT architecture[3], automatically tracking the left ventricular myocardium motion and calculating GLS. In this module, the segmentation network generates the initial endocardial contour in the key frame, which serves as the semantic starting point for subsequent optical flow-based temporal propagation and tracking across the sequence.

The second module was based on the Deeplabv3+[4] architecture to achieve automatic segmentation of the left ventricle and calculate LVEF. In this module, optical flow is utilized to align intermediate frames with the key frames, providing interframe myocardial motion information as temporal context for the segmentation network. This alignment mechanism addresses the challenge of left ventricle segmentation caused by blurry boundaries in individual key frames.

1.5.1 The optical flow module

The optical flow module of Echo-DFCNN was based on the RAFT algorithm[3], optimizing its feature extraction encoder to extract optical flow information between frames in echocardiogram videos, enabling accurate tracking of left ventricular myocardial motion patterns and assessments of GLS. The network consisted of six main components: image feature encoder, context feature encoder, cost volume construction module, cost volume indexing module, iterative update operator, and upsampling module.

**Fig A. Architecture of the optical flow module.**

(1) **Image feature encoder:** This study replaced the original CNN image feature encoder with Twins-SVT[5], encoding images into 256-channel feature maps with an image size of 1/8. It integrated spatial and channel information, improving the model's ability to represent local and global image features, helping to extract more discriminative features, and improving the ability to distinguish the similarity between adjacent frames.
(2) **Context feature encoder:** Due to the lack of semantic information in the image feature encoder's output, which hindered the iterative optimization and upsampling operations of optical flow, a context feature encoder was employed. The context feature encoder comprising six residual modules was used to extract features from the first frame image. The feature map was divided into two parts in the channel dimension, one of which was used as the initial hidden state of the iterative update operator. The other was encoded with the current optical flow and cost volume, serving as the current input to the iterative update operator.
(3) **Cost volume construction module:** Using the features extracted by the image feature encoder to construct a cost volume, and modeling the matching relationship of images. The specific construction method was to perform matrix multiplication on the features between two frames of images, as shown in Formula 1-3, where $f\left(x_{1}\right)$and $f\left(x_{2}\right)$ were the features obtained by the image feature encoder for the first and second frames of images.

$cv\left(x_{1},x_{2}\right)=f\left(x_{1}\right)^{T}f\left(x_{2}\right)$ 1-3

(4) **Cost volume indexing module:** We extracted local cost volumes from the global cost volume according to the current optical flow, namely the "$L$" in **Fig A**, and input them into the iterative update operator. Assuming that the current optical flow is $\left(f^{1},f^{2}\right)$, each pixel in the first frame was transformed to the second frame according to this optical flow, that was, $x'=\left(u+f^{1}\left(u\right),v+f^{2}\left(v\right)\right)$. Then, we defined a local grid centered on $x$, and sampled from the global cost volume by linear interpolation. We sampled the cost volume at four different scales to obtain four local cost volumes, which were then concatenated together in the last dimension.

(5) **Iterative update operator:** This module was designed based on the Gated Recurrent Unit (GRU), and had two inputs, namely the information of the initial hidden state $h_{t-1}$ and the current input $x_{t}$. The information from the initial hidden state was fused with the current input by the update gate and the reset gate. The update gate is $z_{t}$ and the reset gate is $r_{t}$.

In the optical flow module of this study, one output feature of the semantic feature encoder was used as the initial hidden state $h_{t-1}$ of the GRU, while the other output feature of the semantic feature network was encoded with the current optical flow and local cost volume and used as the current input $x_{t}$ of the GRU. Finally, the GRU module obtained the output optical flow by decoding the current state $h_{t}$ through the optical flow decoder.

(6) **Upsampling module:** As the optical flow output by the iterative update operator was a low-resolution optical flow, the upsampling module reconstructed the low-resolution optical flow through convolution operations to obtain a high-resolution optical flow.

1.5.2 The segmentation module

The segmentation module of Echo-DFCNN aims to provide robust and accurate delineation of the left ventricle in keyframes (ED or ES) by leveraging temporal context from adjacent frames. To address the challenge of blurry boundaries and transient degradation in individual frames, we built a pipeline consisting of image-level optical flow alignment, multi-scale feature extraction, attention-based feature fusion, and a segmentation head. The detailed steps of this module are described below.

**Fig B. Architecture of the segmentation module.**

(1) **Optical flow estimation and image-level warping:** The pre-trained optical flow module was employed to estimate the motion fields between adjacent frames. We executed spatial alignment at the image level to preserve fine-grained structural details and explicitly compensate for myocardial motion. Specifically, when predicting a keyframe (denoted as $I_{t}$), its neighboring $k$ ($k=6$) frames were selected. Let $F_{t+j\to t+j-1}$ denote the estimated optical flow from frame $t+j$ to $t+j-1$. The neighboring frames were sequentially warped to the spatial coordinate system of the keyframe. The sequential warping process for an adjacent frame $I_{t+k}$ can be formulated as:

$\tilde{I}_{t+k}=W(\ldots W\left( W\left( I_{t+k},F_{t+j\to t+j-1} \right),F_{t+k-1\to t+k-2} \right),\ldots,F_{t+1\to t})$

where $I_{t+k}$ represents the raw adjacent frame. $W(\cdot,\cdot)$ denotes the differentiable bilinear interpolation function, which resamples the source image according to the flow field. $\tilde{I}_{t+k}$ is the resulting aligned adjacent frame in the coordinate system of the keyframe $I_{t}$.

(2) **Multi-scale feature extraction:** ResNet-34 was employed as the shared feature extractor to capture both detailed spatial information and abstract semantic context. The keyframe $I_{t}$ and the $k$ aligned adjacent frames $\left\{ \tilde{I}_{t+1},\ldots,\tilde{I}_{t+k} \right\}$ were individually inputted into the shared backbone. For each input frame, we extracted multi-scale representations from different intermediate layers of the ResNet-34. Specifically, we obtained low-level feature maps to preserve boundary details, and high-level feature maps to capture global semantic information.
(3) **Attention-based feature fusion:** To effectively aggregate the temporal context from the aligned frames while suppressing potential noise introduced by warping artifacts, a Spatial Attention Gate (SAGate) was introduced. The fusion process was performed independently for both the low-level and high-level feature streams. For a specific feature level, let $f_{t}$ denote the feature map of the keyframe and $\tilde{f}_{t+j}$ denote the feature map of an aligned adjacent frame. The keyframe feature $f_{t}$ was used as the guidance to compute attention weights. First, $f_{t}$ and $\tilde{f}_{t+j}$ were concatenated. A FilterLayer comprising Global Average Pooling (GAP) and a Multi-Layer Perceptron (MLP) was utilized to compute channel-wise dependencies, while $1\times1$ convolutions followed by a Softmax function were applied to generate spatial attention vectors. The input feature maps were then multiplied by these attention vectors to emphasize task-relevant regions. The calibrated features from all $k$ adjacent frames were finally concatenated with the keyframe feature along the channel dimension. This concatenation operation can be expressed as:

$$F_{fused}=Concat(f_{t}, SAGate\left( f_{t}, \tilde{f}_{t+j} \right),\ldots,SAGate\left( f_{t}, \tilde{f}_{t+k} \right))$$

where $F_{fused}$ represents the aggregated feature map containing rich, noise-suppressed temporal context.

(4) **Segmentation head:** We adopted a DeepLabV3+ decoder to process the fused features and generate the final prediction. The fused high-level features were fed into an Atrous Spatial Pyramid Pooling (ASPP) module. By employing parallel atrous convolutions with different dilation rates, the ASPP module effectively captured multi-scale contextual information. To recover spatial details lost during downsampling, the fused low-level features bypassed the ASPP module via a skip connection. After dimensionality reduction via $1\times1$ convolutions, they were concatenated with the upsampled output of the ASPP module. Finally, the concatenated features were decoded by sequential $3\times3$ convolutional layers to produce the segmentation logits for the keyframe.

1.5.3 Loss function

In our proposed two-stage training framework, the loss functions for the optical flow branch and the segmentation branch were designed separately to achieve their respective optimization goals. Both branches utilized a combination of Cross-Entropy (CE) loss and Dice loss, but they were applied to different predictions and targets.

(1) Loss function of optical flow module: In contrast to traditional optical flow networks that rely on dense, pixel-level annotations for supervised learning, our optical flow module was optimized via a cross-frame segmentation consistency loss. Specifically, the ground truth segmentation mask of the starting frame was sequentially warped to the ending frame using the predicted optical flows. Let ${\hat{\boldsymbol{M}}}_{\boldsymbol{w}\boldsymbol{ar}\boldsymbol{p}}$ denote the warped starting mask aligned to the ending frame's coordinate system, and $\boldsymbol{M}_{\boldsymbol{gt}}$ denote the ground truth mask of the ending frame. To further refine the boundary alignment, we also extracted the inner contour mask ($\boldsymbol{M}_{\boldsymbol{in}}$) and outer contour mask ($\boldsymbol{M}_{\boldsymbol{out}}$) from the ground truth. The corresponding warped contour masks are denoted as ${\hat{\boldsymbol{M}}}_{\boldsymbol{warp\_in}}$ and ${\hat{\boldsymbol{M}}}_{\boldsymbol{warp\_out}}$.

The loss for the optical flow module ($\boldsymbol{L}_{\boldsymbol{flow}}$) was formulated as a weighted sum of the main mask consistency loss and the boundary consistency losses:

$$\boldsymbol{L}_{\boldsymbol{flow}}\boldsymbol{=}\boldsymbol{L}_{\boldsymbol{seg}}\left( {\hat{\boldsymbol{M}}}_{\boldsymbol{warp}}\boldsymbol{,}\boldsymbol{M}_{\boldsymbol{gt}} \right)\boldsymbol{+\lambda}\left[ \boldsymbol{L}_{\boldsymbol{seg}}\left( {\hat{\boldsymbol{M}}}_{\boldsymbol{war}\boldsymbol{p}_{\boldsymbol{in}}}\boldsymbol{,}\boldsymbol{M}_{\boldsymbol{in}} \right)\boldsymbol{+}\boldsymbol{L}_{\boldsymbol{seg}}\boldsymbol{(}{\hat{\boldsymbol{M}}}_{\boldsymbol{war}\boldsymbol{p}_{\boldsymbol{out}}}\boldsymbol{,}\boldsymbol{M}_{\boldsymbol{out}}\boldsymbol{)} \right]$$

where λ is a weight coefficient set to 0.05 in our experiments to balance the main region and boundary constraints.

The function $\boldsymbol{L}_{\boldsymbol{seg}}\boldsymbol{(\cdot,\cdot)}$ calculates the segmentation error between the warped prediction $\hat{\boldsymbol{y}}$ and the target $\boldsymbol{y}$, which is defined as:

$$\boldsymbol{L}_{\boldsymbol{seg}}\left( \hat{\boldsymbol{y}}\boldsymbol{,y} \right)\boldsymbol{=}\boldsymbol{L}_{\boldsymbol{CE}}\left( \hat{\boldsymbol{y}}\boldsymbol{,y} \right)\boldsymbol{+}\boldsymbol{L}_{\boldsymbol{Dice}}\left( \hat{\boldsymbol{y}}\boldsymbol{,y} \right)$$

Here, $\boldsymbol{L}_{\boldsymbol{CE}}$ is the standard Cross-Entropy loss, and $\boldsymbol{L}_{\boldsymbol{Dice}}$ is the multi-class Dice loss. Through the differentiable warping operation, the gradients from $\boldsymbol{L}_{\boldsymbol{flow}}$ can be back-propagated to update the parameters of the optical flow module, enabling it to learn myocardial motion patterns without ground truth flow labels.

(2) Loss function of the segmentation module: In the segmentation module training stage, the optical flow module was frozen. The keyframe and its adjacent frames were aligned and fed into the segmentation module to predict the keyframe's segmentation logits. Let ${\hat{\boldsymbol{P}}}_{\boldsymbol{key}}$ denote the predicted probability map of the keyframe, and $\boldsymbol{Y}_{\boldsymbol{key}}$ denote the corresponding ground truth segmentation mask.  The loss function for the segmentation module ($\boldsymbol{L}_{\boldsymbol{segmentation}}$) was also a combination of Cross-Entropy loss and Dice loss, formulated as:

$$\boldsymbol{L}_{\boldsymbol{segmentation}}\boldsymbol{=}\boldsymbol{L}_{\boldsymbol{CE}}\left( {\hat{\boldsymbol{P}}}_{\boldsymbol{key}}\boldsymbol{,}\boldsymbol{Y}_{\boldsymbol{key}} \right)\boldsymbol{+}\boldsymbol{L}_{\boldsymbol{Dice}}\left( {\hat{\boldsymbol{P}}}_{\boldsymbol{key}}\boldsymbol{,}\boldsymbol{Y}_{\boldsymbol{key}} \right)$$

The Cross-Entropy loss ($\boldsymbol{L}_{\boldsymbol{CE}}$) measures the pixel-wise classification error and is calculated as:

$$\boldsymbol{L}_{\boldsymbol{CE}}\boldsymbol{= -}\frac{\boldsymbol{1}}{\boldsymbol{N}}\sum_{\boldsymbol{i=1}}^{\boldsymbol{N}} \sum_{\boldsymbol{c=1}}^{\boldsymbol{C}} \boldsymbol{Y}_{\boldsymbol{key}}^{\boldsymbol{(i,c)}}\boldsymbol{log}\mathbf{}\boldsymbol{(}{\hat{\boldsymbol{P}}}_{\boldsymbol{key}}^{\boldsymbol{(i,c)}}\boldsymbol{)}$$

where $\boldsymbol{N}$ is the total number of pixels in a batch, $\boldsymbol{C}$ is the number of classes (e.g., background, left ventricular cavity, and myocardium), $\boldsymbol{Y}_{\boldsymbol{key}}^{\boldsymbol{(i,c)}}$ is the one-hot encoded ground truth for pixel$\boldsymbol{i}$ belonging to class $\boldsymbol{c}$, and ${\hat{\boldsymbol{P}}}_{\boldsymbol{key}}^{\boldsymbol{(i,c)}}$ is the predicted probability.

The Dice loss ($\boldsymbol{L}_{\boldsymbol{Dice}}$) is utilized to mitigate the issue of class imbalance and focuses on the regional overlap between the prediction and the ground truth. It is calculated as:

$$\boldsymbol{L}_{\boldsymbol{Dice}}\boldsymbol{=}\sum_{\boldsymbol{c=1}}^{\boldsymbol{C}} \boldsymbol{(1-}\frac{\boldsymbol{2}\sum_{\boldsymbol{i=1}}^{\boldsymbol{N}} {\hat{\boldsymbol{P}}}_{\boldsymbol{key}}^{\boldsymbol{(i,c)}}\boldsymbol{Y}_{\boldsymbol{key}}^{\boldsymbol{(i,c)}}}{\sum_{\boldsymbol{i=1}}^{\boldsymbol{N}} {\hat{\boldsymbol{P}}}_{\boldsymbol{key}}^{\boldsymbol{(i,c)}}\boldsymbol{+}\sum_{\boldsymbol{i=1}}^{\boldsymbol{N}} \boldsymbol{Y}_{\boldsymbol{key}}^{\boldsymbol{(i,c)}}}\boldsymbol{)}$$

By optimizing $\boldsymbol{L}_{\boldsymbol{segmentation}}$, the segmentation module learns to leverage the flow-aligned temporal context to produce robust and accurate delineations of the left ventricle in keyframes.

1.5.4 Evaluation of model performance

The accuracy of left ventricular segmentation was evaluated by the Dice similarity coefficient (DSC) and average surface distance (ASD). The accuracy of optical flow tracking of endocardial contours was evaluated by ASD and Hausdorff distance (HD).

DSC was commonly used to calculate the similarity between two images, and was sensitive to the internal filling of the mask. Its calculation formula was:

where $\hat{y}$ represented the prediction and $y$ represented the ground truth.

ASD was used to evaluate the distance between two surfaces (usually the segmentation and the ground truth). For two sets of points $\hat{Y}$ and $Y$, ASD was calculated as follows:

$$ASD(\hat{Y},Y)=\sum_{\hat{y}\in\hat{Y}} \min_{y\in Y}d(\hat{y},y)/\left|\hat{Y}\right|$$

where $\hat{Y}$ represented the prediction and $Y$ represented the ground truth.

$d(\hat{y},y)$ represented the distance from point $\hat{y}$ to point $y$. $\min$ represented the smallest element in the set. $\left|\hat{Y}\right|$ was the length of the set $\hat{Y}$.

HD was a metric that measured the similarity between two sets of points. It was sensitive in comparing segmented boundaries, and was often used to compare the differences between two shapes. For two sets of points $Y$and $\hat{Y}$, the HD was calculated as follows:

$$H(\hat{Y},Y)=\max\left\{\max_{\hat{y}\in\hat{Y}}\left\{\min_{y\in Y}d(\hat{y},y)\right\},\max_{y\in Y}\left\{\min_{\hat{y}\in\hat{Y}}d(\hat{y},y)\right\}\right\}$$

This distance measures the maximum distance between two sets of points. Here, $d(\hat{y},y)$ represented the distance from point $\hat{y}$ to point $y$. $min$represented the smallest element. $max$represented the largest element.

1.6 PRIME checklist

**Table A. Proposed Requirements for Cardiovascular Imaging-Related Machine Learning Evaluation (PRIME) checklist[6]**

| ***Section*** | **Checklist item** | **Section / Figure / Table** |
| --- | --- | --- |
| ***1*** | **Designing the study plan** |  |
| *1.1* | Describe the need for the application of machine learning to the data set | Introduction |
| *1.2* | Describe the objectives of the machine learning analysis | Introduction |
| *1.3* | Define the study plan | Methods – Overview of study design  Fig 1 |
| *1.4* | Describe the summary statistics of baseline data | Results – Clinical characteristics of the study cohorts  Table 2 |
| *1.5* | Describe the overall steps of the machine learning workflow | Methods – Overview of study design  Fig 1 |
| ***2*** | **Data standardization, feature engineering, and learning** |  |
| *2.1* | Describe how the data were processed in order to make it clean, uniform, and consistent | Supplemental Methods 1.2 in S1 Appendix |
| *2.2* | Describe whether variables were normalized and, if so, how this was done | Supplemental Methods 1.2 and 1.3 in S1 Appendix |
| *2.3* | Provide details on the fraction of missing values (if any) and imputation methods | N/A |
| *2.4* | Perform and describe the feature selection process | Methods – Model  Supplemental Methods 1.5 in S1 Appendix |
| *2.5* | Identify and describe the process to handle outliers, if any | N/A |
| *2.6* | Describe whether class imbalance existed and which method was applied to deal with it | N/A |
| ***3*** | **Selection of a machine learning model** |  |
| *3.1* | Explicitly define the goal of the analysis, e.g., regression, classification, clustering | Methods – Overview of study design |
| *3.2* | Identify the proper learning method used (e.g., supervised, reinforcement learning, etc.) to address the problem | Methods – Model  Supplemental Methods 1.5 in S1 Appendix |
| *3.3* | Provide explicit details on the use of simpler, complex, or ensemble models | N/A |
| *3.4* | Provide the comparison of complex models against simpler models if possible | N/A |
| *3.5* | Define ensemble methods, if used | Methods – Model  Supplemental Methods 1.5 in S1 Appendix  Figs A and B in S1 Appendix |
| *3.6* | Provide details on whether the model is interpretable | Fig 1  Discussion  Fig 6 |

**Table A. Continued**

| ***Section*** | **Checklist item** | **Section / Figure / Table** |
| --- | --- | --- |
| ***4*** | **Model assessment** |  |
| *4.1* | Provide a clear description of data used for training, validation, and testing | Methods – Datasets  Fig 2  Supplemental Methods 1.4 in S1 Appendix |
| *4.2* | Describe how the model parameters were optimized (e.g., optimization technique, number of model parameters, etc.) | Methods – Model  Supplemental Methods 1.5 in S1 Appendix |
| ***5*** | **Model evaluation** |  |
| *5.1* | Provide the metric(s) used to evaluate the performance of the model | Methods – Statistical analysis  Supplemental Methods 1.5.4 in S1 Appendix |
| *5.2* | Define the prevalence of disease and the choice of the scoring rule used | N/A |
| *5.3* | Report any methods used to balance the numbers of subjects in each class | N/A |
| *5.4* | Discuss the risk associated to misclassification | Failure to identify left ventricular systolic dysfunction early may lead to further deterioration in cardiac function and ultimately develop into heart failure. |
| ***6*** | **Best practices for model replicability** |  |
| *6.1* | Consider sharing code or scripts in a public repository with appropriate copyright protection steps for further development and non-commercial use | https://github.com/yz-hust/Echo-DFCNN |
| *6.2* | Release data dictionary with appropriate explanation of the variables | https://kaggle.com/datasets/0d99e1782aee1a92fdacaea03a5256d9169aacd515ea4535eada050175c9b2ea |
| *6.3* | Document version of all software and external libraries | Methods – Statistical analysis |
| ***7*** | **Reporting limitations, biases, and alternatives** |  |
| *7.1* | Identify and report the relevant model assumptions and findings | Methods – Model  Results  Supplemental Methods 1.5 in S1 Appendix  Supplemental Methods 2 in S1 Appendix |
| *7.2* | If well-performing models were tested on a hold-out validation data set, detail the data of that validation set with the same rigor as that of the training data set (see section 2 above) | Methods – Datasets  Fig 4  Table 3  Results –Generalization to external medical centers |

2. SUPPLEMENTAL RESULTS

2.1 Model performance

**Table B. Segmentation performance of the left ventricle on the internal test dataset (n=100) and external validation datasets (n=363)**

|  | | A2C | | |  | A3C | | |  | A4C | |
| --- | --- | --- | --- | --- | --- | --- | --- | --- | --- | --- | --- |
|  | ED | | ES |  | | ED | ES |  | | ED | ES |
| Internal test dataset |  | |  |  | |  |  |  | |  |  |
| DSC, mean (SD) | 0.95 (0.02) | | 0.93 (0.05) |  | | 0.94 (0.03) | 0.92 (0.05) |  | | 0.95 (0.03) | 0.94 (0.05) |
| ASD, mean (SD), mm | 1.80 (0.65) | | 1.90 (0.87) |  | | 1.83 (0.67) | 1.97 (0.94) |  | | 1.71 (0.85) | 1.72 (1.02) |
| External dataset 1 |  | |  |  | |  |  |  | |  |  |
| DSC, mean (SD) | 0.90 (0.05) | | 0.88 (0.06) |  | | 0.90 (0.05) | 0.88 (0.06) |  | | 0.91 (0.05) | 0.89 (0.06) |
| ASD, mean (SD), mm | 2.02 (0.75) | | 1.92 (0.68) |  | | 2.22 (3.01) | 2.13 (1.40) |  | | 2.79 (6.30) | 2.27 (2.87) |
| External dataset 2 |  | |  |  | |  |  |  | |  |  |
| DSC, mean (SD) | 0.85 (0.05) | | 0.80 (0.07) |  | | 0.86 (0.04) | 0.80 (0.07) |  | | 0.86 (0.05) | 0.80 (0.10) |
| ASD, mean (SD), mm | 2.13 (0.80) | | 2.30 (0.98) |  | | 2.10 (0.71) | 2.52 (1.15) |  | | 2.15 (1.53) | 2.21 (1.68) |
| External dataset 3 |  | |  |  | |  |  |  | |  |  |
| DSC, mean (SD) | 0.86 (0.08) | | 0.80 (0.15) |  | | 0.84 (0.11) | 0.79 (0.14) |  | | 0.87 (0.07) | 0.82 (0.11) |
| ASD, mean (SD), mm | 1.51 (1.39) | | 1.82 (3.25) |  | | 1.79 (2.45) | 1.68 (1.35) |  | | 1.34 (1.04) | 1.84 (3.68) |
| External dataset 4 |  | |  |  | |  |  |  | |  |  |
| DSC, mean (SD) | 0.83 (0.03) | | 0.79 (0.05) |  | | 0.85 (0.03) | 0.80 (0.04) |  | | 0.85 (0.03) | 0.81 (0.05) |
| ASD, mean (SD), mm | 1.69 (0.54) | | 1.71 (0.51) |  | | 1.67 (0.61) | 1.76 (0.76) |  | | 1.54 (0.49) | 1.51 (0.62) |

A2C, apical 2-chamber view; A3C, apical 3-chamber view; A4C, apical 4-chamber view; DSC, Dice similarity coefficient; ASD, average surface distance; ED, end-diastolic; ES, end-systolic; SD, standard deviation.

**Table C. Optical flow tracking performance of the left ventricle on the internal test dataset (n=100) and external validation datasets (n=363)**

|  | A2C | | | A3C | | A4C |
| --- | --- | --- | --- | --- | --- | --- |
| Internal test dataset | |  |  | |  | |
| HD, mean (SD), mm | | 5.72 (3.45) | 6.55 (3.43) | | 5.20 (2.94) | |
| ASD, mean (SD), mm | | 2.05 (0.76) | 2.19 (0.83) | | 1.97 (0.93) | |
| External dataset 1 | |  |  | |  | |
| HD, mean (SD), mm | | 5.84 (3.69) | 9.24 (11.80) | | 7.51 (9.30) | |
| ASD, mean (SD), mm | | 1.98 (0.71) | 2.42 (2.49) | | 2.83 (5.36) | |
| External dataset 2 | |  |  | |  | |
| HD, mean (SD), mm | | 7.51 (5.57) | 6.70 (3.60) | | 6.93 (5.06) | |
| ASD, mean (SD), mm | | 2.39 (0.93) | 2.34 (0.92) | | 2.47 (1.63) | |
| External dataset 3 | |  |  | |  | |
| HD, mean (SD), mm | | 5.76 (7.11) | 7.80 (10.64) | | 5.34 (6.80) | |
| ASD, mean (SD), mm | | 1.77 (1.69) | 2.27 (3.51) | | 1.71 (1.36) | |
| External dataset 4 | |  |  | |  | |
| HD, mean (SD), mm | | 5.83 (1.76) | 5.79 (1.76) | | 5.92 (4.46) | |
| ASD, mean (SD), mm | | 2.21 (0.81) | 1.97 (0.63) | | 1.92 (0.73) | |

A2C, apical 2-chamber view; A3C, apical 3-chamber view; A4C, apical 4-chamber view; HD, Hausdorff distance; ASD, average surface distance; ED, end-diastolic; ES, end-systolic; SD, standard deviation.

2.2 Assessment of cardiac function

**Table D. Measurements of LVEF and GLS on the internal test dataset (n=100) and external validation datasets (n=363)**

|  | AI | Manual |
| --- | --- | --- |
|  |  |  |
| Internal test dataset |  |  |
| LVEDV, median (IQR), ml | 144.50 (89.27, 202.61) | 134.42 (89.87, 198.87) |
| LVESV, median (IQR), ml | 91.95 (41.89, 135.32) | 97.30 (43.80, 137.95) |
| LVEF, median (IQR), % | 37.67 (27.93, 50.89) | 36.44 (27.30, 53.37) |
| GLS, median (IQR), % | -13.90 (-18.08, -9.87) | -12.33 (-17.74, -9.65) |
| External dataset 1 |  |  |
| LVEDV, median (IQR), ml | 104.85 (84.56, 144.36) | 103.41 (83.24, 136.37) |
| LVESV, median (IQR), ml | 49.70 (35.76, 87.94) | 46.87 (35.94, 80.54) |
| LVEF, median (IQR), % | 49.66 (37.38, 58.68) | 51.58 (37.70, 58.07) |
| GLS, median (IQR), % | -16.99 (-20.73, -12.34) | -16.41 (-19.62, -11.89) |
| External dataset 2 |  |  |
| LVEDV, median (IQR), ml | 87.16 (75.19, 101.04) | 86.89 (76.81, 101.88) |
| LVESV, median (IQR), ml | 35.20 (28.73, 44.06) | 33.67 (27.16, 44.25) |
| LVEF, median (IQR), % | 59.02 (51.77, 65.04) | 59.18 (53.10, 66.51) |
| GLS, median (IQR), % | -21.42 (-23.66, -19.37) | -21.91 (-24.54, -18.94) |
| External dataset 3 |  |  |
| LVEDV, median (IQR), ml | 94.21 (75.95, 123.39) | 95.08 (68.20, 120.00) |
| LVESV, median (IQR), ml | 39.66 (26.71, 66.26) | 37.10 (24.14, 68.10) |
| LVEF, median (IQR), % | 55.86 (41.30, 63.89) | 57.88 (43.61, 64.14) |
| GLS, median (IQR), % | -20.40 (-23.29, -16.52) | -20.12 (-23.47, -15.75) |
| External dataset 4 |  |  |
| LVEDV, median (IQR), ml | 93.58 (69.32, 107.48) | 92.11 (66.46, 103.37) |
| LVESV, median (IQR), ml | 35.13 (27.61, 48.32) | 34.53 (26.19, 48.35) |
| LVEF, median (IQR), % | 59.08 (52.11, 63.10) | 58.35 (54.81, 62.97) |
| GLS, median (IQR), % | -20.85 (-24.73, -18.26) | -21.84 (-23.30, -18.37) |

LVEDV, left ventricular end-diastolic volume; LVESV, left ventricular end-systolic volume; LVEF, left ventricular ejection fraction; GLS, global longitudinal strain; SD, standard deviation; IQR, interquartile range.

**Table E. Stratified Bland–Altman agreement for LVEF and GLS across different cardiac function ranges on the internal test dataset (n = 100).**

|  | n | Bias | SD of differences | LOA | LOA width |
| --- | --- | --- | --- | --- | --- |
| LVEF, % |  |  |  |  |  |
| LVEF < 30% | 25 | -0.40 | 3.90 | [-8.05, 7.25] | 15.30 |
| LVEF 30-39% | 25 | 2.10 | 4.70 | [-7.12, 11.32] | 18.44 |
| LVEF 40-49% | 25 | 2.67 | 3.01 | [-3.23, 8.57] | 11.80 |
| LVEF ≥ 50% | 25 | -0.81 | 9.75 | [-19.91, 18.29] | 38.20 |
| GLS, % |  |  |  |  |  |
| LVEF < 30% | 25 | -0.22 | 1.49 | [-3.14, 2.70] | 5.85 |
| LVEF 30-39% | 25 | -1.14 | 1.78 | [-4.63, 2.35] | 6.98 |
| LVEF 40-49% | 25 | -1.14 | 1.28 | [-3.63, 1.36] | 5.00 |
| LVEF ≥ 50% | 25 | -2.37 | 2.66 | [-7.59, 2.85] | 10.44 |

Bias is defined as AI minus manual measurement. LOA indicates the 95% limits of agreement (bias ± 1.96 × SD of differences).

**Fig C. Classification of reduced ejection fraction on the test dataset (n=100).**

2.3 Agreement categorized using LVEF, image quality, and machine types

**Table F. Measurements of LVEF and GLS in the subgroup of the internal test dataset (n=100) by LVEF ranges**

|  |  | LVEF<30%  (n=25) | LVEF 30-39%  (n=25) | LVEF 40-49%  (n=25) | LVEF≥50%  (n=25) |
| --- | --- | --- | --- | --- | --- |
| AI | LVEF, median (IQR), % | 23.61  (17.54, 27.60) | 34.35  (29.09, 37.77) | 44.50  (38.85, 46.32) | 62.04  (56.00, 65.73) |
|  | GLS, median (IQR), % | -7.85  (-12.18, -5.95) | -13.08  (-13.95, -8.95) | -15.60 (-17.67, -12.71) | -22.23 (-25.22, -19.50) |
| Manual | LVEF, median (IQR), % | 23.14  (17.16, 27.42) | 31.60  (27.60, 36.44) | 40.84  (36.32, 44.75) | 59.85  (56.86, 67.11) |
|  | GLS, median (IQR), % | -8.49  (-10.82, -6.54) | -10.52  (-12.30, -9.24) | -13.97 (-16.19, -12.22) | -20.00 (-21.31, -18.47) |

LVEF, left ventricular ejection fraction; GLS, global longitudinal strain; IQR, interquartile range.

**Table G. Correlation and agreement between AI and manual measurements of LVEF and GLS in the subgroup of the internal test dataset (n=100) by LVEF ranges**

|  |  | LVEF<30%  (n=25) | LVEF 30-39%  (n=25) | LVEF 40-49%  (n=25) | LVEF≥50%  (n=25) |
| --- | --- | --- | --- | --- | --- |
| Median absolute error, (IQR) | LVEF, % | 2.68 (0.89, 3.96) | 3.12 (2.15, 5.47) | 3.09 (1.31, 4.85) | 3.66 (1.47, 6.44) |
|  | GLS, % | 1.28 (0.35, 1.79) | 1.26 (0.66, 2.93) | 0.97 (0.52, 1.65) | 2.96 (1.66, 4.77) |
| Mean absolute error, (SD) | LVEF, % | 2.96 (2.51) | 4.09 (3.04) | 3.28 (2.29) | 5.45 (8.04) |
|  | GLS, % | 1.21 (0.87) | 1.72 (1.20) | 1.32 (1.07) | 3.07 (1.76) |
| r, (p-value) | LVEF, % | 0.853  (p < 0.0001) | 0.661  (p < 0.001) | 0.828  (p < 0.0001) | 0.632  (p < 0.001) |
|  | GLS, % | 0.935  (p < 0.0001) | 0.769  (p < 0.0001) | 0.907  (p < 0.0001) | 0.618  (p = 0.001) |
| ICC, [95% CI] | LVEF, % | 0.863  [0.715, 0.937] | 0.659  [0.353, 0.835] | 0.817  [0.335, 0.936] | 0.304  [-0.106, 0.622] |
|  | GLS, % | 0.898  [0.784, 0.954] | 0.677  [0.299, 0.856] | 0.871  [0.450, 0.956] | 0.637  [0.102, 0.854] |

LVEF, left ventricular ejection fraction; GLS, global longitudinal strain; IQR, interquartile range; SD, standard deviation; r, Spearman correlation coefficient; CI, confidence interval; ICC, intraclass correlation coefficients.

**Table H. Correlation and agreement between AI and manual measurements of LVEF in the subgroup of the internal test dataset (n=100) and external validation datasets (n=363) by image quality**

|  | Good  (n=56) | Fair  (n=374) | Poor  (n=33) |
| --- | --- | --- | --- |
| Median absolute error, (IQR) | 2.90 (1.60, 5.28) | 3.52 (1.41, 6.44) | 3.66 (2.07, 6.14) |
| Mean absolute error, (SD) | 4.23 (3.68) | 4.69 (4.81) | 4.90 (4.53) |
| r, (p-value) | 0.919 (p < 0.0001) | 0.858 (p < 0.0001) | 0.740 (p < 0.0001) |
| ICC, [95% CI] | 0.931 [0.886, 0.959] | 0.896 [0.874, 0.915] | 0.850 [0.706, 0.924] |

IQR, interquartile range; SD, standard deviation; r, Spearman correlation coefficient; CI, confidence interval; ICC, intraclass correlation coefficients.

**Table I. Correlation and agreement between AI and manual measurements of GLS in the subgroup of the internal test dataset (n=100) and external validation datasets (n=363) by image quality**

|  | Good  (n=65) | Fair  (n=366) | Poor  (n=32) |
| --- | --- | --- | --- |
| Median absolute error, (IQR) | 1.30 (0.59, 2.29) | 1.67 (0.75, 3.15) | 1.58 (0.72, 3.55) |
| Mean absolute error, (SD) | 1.60 (1.27) | 2.19 (2.01) | 2.69 (3.14) |
| r, (p-value) | 0.934 (p < 0.0001) | 0.830 (p < 0.0001) | 0.503 (p < 0.01) |
| ICC, [95% CI] | 0.938 [0.900, 0.962] | 0.859 [0.829, 0.883] | 0.596 [0.315, 0.780] |

IQR, interquartile range; SD, standard deviation; r, Spearman correlation coefficient; CI, confidence interval; ICC, intraclass correlation coefficients.

**Table J. Sensitivity analysis of AI and manual LVEF measurements across image quality subgroups using stricter categorical thresholds (+1 point shift)**

|  | Good  (n=29) | Fair  (n=373) | Poor  (n=61) |
| --- | --- | --- | --- |
| Median absolute error, (IQR) | 2.75 (1.31, 4.40) | 3.32 (1.48, 6.25) | 4.28 (2.23, 7.16) |
| Mean absolute error, (SD) | 3.59 (3.27) | 4.50 (4.43) | 6.09 (6.18) |
| r, (p-value) | 0.934 (p = 0.0046) | 0.868 (p < 0.0001) | 0.745 (p < 0.0001) |
| ICC, [95% CI] | 0.942 [0.882, 0.972] | 0.909 [0.890, 0.926] | 0.786 [0.658, 0.869] |

IQR, interquartile range; SD, standard deviation; r, Spearman correlation coefficient; CI, confidence interval; ICC, intraclass correlation coefficients. For LVEF: The Kruskal-Wallis H-test with Benjamini-Hochberg correction revealed no significant overall difference among image quality subgroups (adjusted p = 0.138).

**Table K. Sensitivity analysis of AI and manual GLS measurements across image quality subgroups using stricter categorical thresholds (+1 point shift).**

|  | Good  (n=44) | Fair  (n=366) | Poor  (n=53) |
| --- | --- | --- | --- |
| Median absolute error, (IQR) | 1.29 (0.63, 2.35) | 1.67 (0.75, 3.09) | 1.48 (0.67, 3.44) |
| Mean absolute error, (SD) | 1.59 (1.28) | 2.15 (1.93) | 2.59 (2.97) |
| r, (p-value) | 0.932 (p < 0.0001) | 0.847 (p < 0.0001) | 0.579 (p < 0.0001) |
| ICC, [95% CI] | 0.934 [0.882, 0.963] | 0.871 [0.844, 0.894] | 0.626 [0.430, 0.766] |

IQR, interquartile range; SD, standard deviation; r, Spearman correlation coefficient; CI, confidence interval; ICC, intraclass correlation coefficients. For GLS: The Kruskal-Wallis H-test with Benjamini-Hochberg correction revealed no significant overall difference among image quality subgroups (adjusted p = 0.341).

**Table L. Sensitivity analysis of AI and manual LVEF measurements across image quality subgroups using looser categorical thresholds (-1 point shift).**

|  | Good  (n=92) | Fair  (n=349) | Poor  (n=22) |
| --- | --- | --- | --- |
| Median absolute error, (IQR) | 2.90 (1.50, 6.12) | 3.58 (1.51, 6.38) | 3.63 (2.43, 6.68) |
| Mean absolute error, (SD) | 4.14 (3.54) | 4.75 (4.90) | 5.25 (4.97) |
| r, (p-value) | 0.931 (p < 0.0001) | 0.848 (p < 0.0001) | 0.673 (p < 0.001) |
| ICC, [95% CI] | 0.939 [0.910, 0.959] | 0.890 [0.866, 0.910] | 0.858 [0.689, 0.938] |

IQR, interquartile range; SD, standard deviation; r, Spearman correlation coefficient; CI, confidence interval; ICC, intraclass correlation coefficients. For LVEF: The Kruskal-Wallis H-test with Benjamini-Hochberg correction revealed no significant overall difference among image quality subgroups (adjusted p = 0.705).

**Table M. Sensitivity analysis of AI and manual GLS measurements across image quality subgroups using looser categorical thresholds (-1 point shift).**

|  | Good  (n=98) | Fair  (n=339) | Poor  (n=26) |
| --- | --- | --- | --- |
| Median absolute error, (IQR) | 1.28 (0.57, 2.45) | 1.73 (0.77, 3.16) | 1.93 (0.67, 3.74) |
| Mean absolute error, (SD) | 1.62 (1.30) | 2.23 (2.05) | 3.04 (3.39) |
| r, (p-value) | 0.925 (p < 0.0001) | 0.827 (p < 0.0001) | 0.450 (p < 0.05) |
| ICC, [95% CI] | 0.935 [0.904, 0.956] | 0.854 [0.822, 0.880] | 0.556 [0.220, 0.774] |

IQR, interquartile range; SD, standard deviation; r, Spearman correlation coefficient; CI, confidence interval; ICC, intraclass correlation coefficients. For GLS: The Kruskal-Wallis H-test with Benjamini-Hochberg correction revealed no significant overall difference among image quality subgroups (adjusted p = 0.091).

**Table N. Correlation and agreement between AI and manual measurements of LVEF and GLS in the subgroup of the internal test dataset (n=100) and external validation datasets (n=363) by machine types**

|  |  | EPIQ 7C  (n=187) | EPIQ 5  (n=38) | EPIQ CVx  (n=29) | iE33  (n=16) | Vivid E95  (n=76) | Vivid E9  (n=72) | LISENDO 880  (n=18) |
| --- | --- | --- | --- | --- | --- | --- | --- | --- |
| Median absolute error, (IQR) | LVEF, % | 3.93 (1.73, 6.22) | 2.42 (1.38, 6.38) | 2.82 (1.19, 5.10) | 2.66 (1.02, 5.28) | 2.68 (1.30, 5.20) | 4.58 (2.25, 9.64) | 2.17 (0.65, 6.03) |
|  | GLS, % | 1.64 (0.73, 3.22) | 2.30 (0.99, 3.55) | 1.58 (0.80, 2.41) | 0.71 (0.34, 1.90) | 1.69 (0.55, 2.90) | 1.46 (0.73, 2.61) | 1.00 (0.52, 1.56) |
| Mean absolute error, (SD) | LVEF, % | 5.09 (5.63) | 3.83 (3.44) | 3.38 (2.66) | 3.56 (3.00) | 3.51 (2.67) | 6.28 (5.25) | 3.33 (3.29) |
|  | GLS, % | 2.29 (2.26) | 2.50 (1.90) | 2.08 (2.25) | 1.37 (1.38) | 2.13 (2.13) | 1.92 (1.62) | 1.23 (1.10) |
| r, (p-value) | LVEF, % | 0.872 (p < 0.0001) | 0.929 (p < 0.0001) | 0.833 (p < 0.0001) | 0.915 (p < 0.0001) | 0.891 (p < 0.0001) | 0.752 (p < 0.0001) | 0.860 (p < 0.0001) |
|  | GLS, % | 0.855 (p < 0.0001) | 0.847 (p < 0.0001) | 0.617 (p < 0.001) | 0.894 (p < 0.0001) | 0.870 (p < 0.0001) | 0.811 (p < 0.0001) | 0.905 (p < 0.0001) |
| ICC, [95% CI] | LVEF, % | 0.907 [0.878, 0.929] | 0.935 [0.878, 0.965] | 0.860 [0.723, 0.932] | 0.940 [0.841, 0.978] | 0.926 [0.886, 0.952] | 0.754 [0.502, 0.868] | 0.961 [0.896, 0.985] |
|  | GLS, % | 0.879 [0.841, 0.908] | 0.839 [0.660, 0.921] | 0.500 [0.180, 0.727] | 0.923 [0.795, 0.972] | 0.838 [0.693, 0.908] | 0.832 [0.744, 0.891] | 0.956 [0.876, 0.984] |

LVEF, left ventricular ejection fraction; GLS, global longitudinal strain; IQR, interquartile range; SD, standard deviation; r, Spearman correlation coefficient; CI, confidence interval; ICC, intraclass correlation coefficients. Machine types with sample sizes ≤15 were excluded from analysis.

2.4 In between methods agreement AI vs. commercial software QLAB

**Table O. Correlation and agreement between the AI-predicted and QLAB measurements of LVEF and GLS on the internal test dataset (n=100) and external validation datasets (n=363)**

|  | Correlation | | |  | Agreement | | |
| --- | --- | --- | --- | --- | --- | --- | --- |
|  | | r | p-value |  | Bias | LOA | ICC, [95% CI] |
| LVEF, % | | 0.850 | p < 0.0001 |  | -2.60 | [-17.04, 11.84] | 0.858 [0.804, 0.894] |
| GLS, % | | 0.814 | p < 0.0001 |  | 0.50 | [-6.14, 7.14] | 0.831 [0.800, 0.858] |

LVEF, left ventricular ejection fraction; GLS, global longitudinal strain; r, Spearman correlation coefficient; CI, confidence interval; LOA, limits of agreement; ICC, intraclass correlation coefficients.

**Fig D. Correlation and agreement between the AI-predicted and QLAB measurements of LVEF and GLS (n=463).** **A,** Correlation between the AI-predicted and QLAB measurements of LVEF and GLS. **B,** Agreement between the AI-predicted and QLAB measurements of LVEF and GLS.

**Table P. Comparison of time consumption between AI and QLAB on the internal test dataset (n=100) and external validation datasets (n=363)**

| Method | Component | Time, median (IQR), seconds | |
| --- | --- | --- | --- |
| AI | Model inference | 11.57 (10.87, 12.41) |  |
| QLAB | User Interaction | 394.41 (345.91, 455.95) |  |
|  | Software Computation | 15.76 (14.44, 17.10) | |
|  | Total | 411.11 (362.53, 471.06) | |

QLAB measured LVEF using the function of a2DQ and GLS using the function of AutoStrain LV. QLAB time consumption did not include data loading time, but only included user interaction time and software computation time. AI time consumption did not include data loading and model loading time, but only referred to the AI model inference time.

2.5 Intra- and interobserver variability and agreement

**Table Q. Inter-observer and intra-observer variability analysis (n=50)**

|  | Agreement | | |
| --- | --- | --- | --- |
|  | Bias | LOA | ICC, [95% CI] |
| LVEF, % |  |  |  |
| Intra-observer | -0.89 | [-4.95, 3.17] | 0.986 [0.971, 0.993] |
| Inter-observer | -2.80 | [-8.97, 3.37] | 0.951 [0.748, 0.982] |
| GLS, % |  |  |  |
| Intra-observer | 0.29 | [-1.55, 2.13] | 0.975 [0.955, 0.986] |
| Inter-observer | 0.92 | [-1.78, 3.62] | 0.932 [0.801, 0.970] |

LVEF, left ventricular ejection fraction; GLS, global longitudinal strain; LOA, limits of agreement; ICC, intraclass correlation coefficients; CI, confidence interval.

**Fig E. Inter-observer and intra-observer variability of LVEF and GLS measured manually (n=50).** **A,** Inter-observer and intra-observer variability of LVEF measured manually by one observer and two observers. **B,** Inter-observer and intra-observer variability of GLS measured manually by one observer and two observers.

2.6 Cross-modal validation of Echo-DFCNN using CMR

**Table R. Clinical stability assessment between Echocardiography and CMR examinations on the CMR validation dataset (n=67)**

|  | CMR validation dataset (n=67) |
| --- | --- |
|  |  |
| Hospitalization between examinations, n (%) |  |
| Inpatient for both scans | 56 (83.6) |
| Outpatient for both scans | 11 (16.4) |
| Admission to the hospital between scans | 0 (0.0) |
| Clinical events between examinations, n (%) |  |
| Cardiac surgery | 0 (0.0) |
| Percutaneous coronary intervention | 0 (0.0) |
| Cardiac implantable electronic device implantation | 0 (0.0) |
| Catheter ablation | 0 (0.0) |
| Acute coronary syndrome | 0 (0.0) |

**Table S. Measurements of LVEF and GLS using different techniques on the CMR validation dataset (n=67)**

|  | AI | QLAB | CMR |
| --- | --- | --- | --- |
| LVEF, median (IQR), % | 20.78 (12.82, 26.46) | 22.90 (18.20, 27.60) | 16.74 (12.33, 20.73) |
| GLS, median (IQR), % | -8.36 (-10.63, -6.37) | -8.17 (-10.53, -6.73) | -8.04 (-9.83, -6.21) |

LVEF, left ventricular ejection fraction; GLS, global longitudinal strain; IQR, interquartile range.

2.7 Computational benchmarking of Echo-DFCNN

**Table T. Computational performance of Echo-DFCNN under different hardware configurations (n=25)**

| Configuration | Inference time, median (IQR), seconds | Successful cases | |
| --- | --- | --- | --- |
| GPU: NVIDIA GeForce RTX 3090 with 4 helper CPU threads | 14.41 (14.03, 15.71) | 25/25 |  |
| CPU: Intel Xeon Silver 4216, 32 cores | 1259.79 (1178.09, 1403.50) | 25/25 |  |
| CPU: Intel Xeon Silver 4216, 16 cores | 1948.58 (1831.46, 2169.39) | 25/25 |  |
| CPU: Intel Xeon Silver 4216, 8 cores | 3383.87 (3207.57, 3780.04) | 25/25 |  |

Echo-DFCNN consists of a segmentation network with 34.33 million parameters and an optical-flow network with 8.47 million parameters, yielding a total of 42.80 million parameters and an FP32 parameter footprint of approximately 163.28 MiB. All tests were performed on a single workstation in a single-process setting after model loading and warm-up. Two repeats were conducted for each condition, with the order of the hardware configurations reversed in the second repeat to mitigate potential order effects. Inference time did not include data preprocessing, network transfer, or concurrent-user workloads.

2.8 The ablation study

**Table U. Comparison of errors in LVEF measurements between Single-seg and Assisted-seg** **on the internal test dataset (n=100) and** **external validation datasets (n=363)**

| Parameter | Dataset | Strategy | Absolute error (AI vs. Manual) | |
| --- | --- | --- | --- | --- |
|  |  |  | Median, (IQR), % | Mean, (SD), % |
| LVEF | Internal test dataset | Single-seg | 3.20 (1.71, 5.66) | 4.81 (6.51) |
|  |  | Assisted-seg | 3.02 (1.54, 4.95) | 3.94 (4.66) |
|  | External dataset 1 | Single-seg | 4.05 (1.66, 5.56) | 4.41 (3.45) |
|  |  | Assisted-seg | 2.80 (1.23, 5.37) | 3.62 (3.02) |
|  | External dataset 2 | Single-seg | 5.46 (2.84, 9.46) | 6.95 (6.00) |
|  |  | Assisted-seg | 4.35 (2.05, 8.13) | 5.82 (5.11) |
|  | External dataset 3 | Single-seg | 4.44 (2.14, 7.70) | 5.50 (4.51) |
|  |  | Assisted-seg | 4.11 (1.66, 6.68) | 5.39 (5.68) |
|  | External dataset 4 | Single-seg | 3.06 (1.89, 4.98) | 3.65 (2.74) |
|  |  | Assisted-seg | 2.39 (1.07, 5.11) | 3.05 (2.39) |

Single-seg, segmentation without optical flow network assistance; Assisted-seg, segmentation with optical flow network assistance; LVEF, left ventricular ejection fraction; IQR, interquartile range; SD, standard deviation. Note that two cases (one from external dataset 2 and one from external dataset 3) were excluded from the analysis due to Single-seg method failure.

**Fig F. Comparison of errors in LVEF measurements between Single-seg (blue) and Assisted-seg (red) on the internal test dataset (n=100) and external validation datasets (n=363) by image quality.** In the box plot, the horizontal line inside the box represents the median, and the square represents the mean. The lower and upper borders of the box represent the lower and upper quartiles (25th and 75th percentiles), respectively. The whiskers extend up to a maximum of 1.5 IQR beyond the upper (Q3) and lower (Q1) quartiles. Individual dots represent outlier values. Note that one case from external dataset 2 (in the fair-quality group) and one case from external dataset 3 (in the poor-quality group) were excluded from the analysis due to Single-seg method failure. Interquartile range, IQR.

**Fig G. Visualization of two cases demonstrating segmentation failure of the Single-seg method. A,** A case from external dataset 2 (fair-quality group), where the Single-seg method exhibited significant segmentation error in the apical 4-chamber (A4C) view at end-diastole (ED). **B,** A case from external dataset 3 (poor-quality group), where the Single-seg method showed significant segmentation error in the apical 2-chamber (A2C) view at end-diastole (ED). In both cases, the segmentation errors of the Single-seg method led to a predicted LVEDV smaller than the LVESV, consequently resulting in a negative LVEF. In contrast, the Assisted-seg method, aided by the optical flow network, significantly improved segmentation accuracy.

**Table V. Comparison of model inference time in LVEF measurements between Single-seg and Assisted-seg on the internal test dataset (n=100) and external validation datasets (n=363)**

| Method | Time, median (IQR), seconds | |
| --- | --- | --- |
| Single-seg | 2.10 (2.09, 2.19) |  |
| Assisted-seg | 3.93 (3.75, 4.14) | |

Single-seg, segmentation without optical flow network assistance; Assisted-seg, segmentation with optical flow network assistance; IQR, interquartile range. Note that one case from external dataset 2 and one case from external dataset 3 were excluded from the analysis due to Single-seg method failure.

3. SUPPLEMENTAL DISCUSSION

**Table W. Studies of AI Application to LVEF and GLS**

| Authors | Year | Task | Model Architecture | Dataset | Main Metrics | Results |
| --- | --- | --- | --- | --- | --- | --- |
| Ouyang et al[7] | 2020 | LVEF assessment (A4C, single-plane Simpson's method) | EchoNet-Dynamic: DeepLabv3 with R2+1D spatiotemporal convolutions | EchoNet-Dynamic: 10,030 videos (USA); The external test dataset: 2,895 videos (USA) | Dice, MAE | DSC (A4C: 0.92); MAE: 4.1% (Internal dataset), 6.0% (External dataset) |
| Tromp J et al[8] | 2022 | LVEF assessment (A2C and A4C, biplane Simpson's method) | U-Net | ATTRaCT: 1,551 echocardiograms from 1482 patients (Asia, 11 countries); Alberta HEART study: 1,029 echocardiograms from 621 patients (Canada); Mackay Memorial Hospital: 31 241 echocardiograms from 9,289 patients (Taiwan); EchoNet-Dynamic: 10,030 videos (USA) | MAE, Correlation coefficient | MAE: 5.5% (ATTRaCT), 8.6% (Alberta HEART study), 10.2% (Mackay Memorial Hospital), 6.5% (EchoNet-Dynamic); r: 0.89 (ATTRaCT), 0.75 (Alberta HEART study), 0.75 (Mackay Memorial Hospital), 0.76 (EchoNet-Dynamic) |
| Dai W et al[9] | 2023 | LVEF assessment (A4C, single-plane Simpson's method) | CSS: DeepLabv3 for LV segmentation; R2+1D ResNet for LVEF regression | EchoNet-Dynamic: 10,030 videos (USA); CAMUS: 450 patients (France) | MAE | MAE: 4.17% (EchoNet-Dynamic), 6.11% (CAMUS) |
| Li H et al[10] | 2023 | LVEF assessment (A2C and A4C, biplane Simpson's method) | EchoEFNet: ResNet-50 with dilated convolution | CAMUS: 450 patients (France); CMUEcho: 191 patients (China) | DSC, Correlation coefficient, ICC | DSC: CAMUS (A2C: 0.927, A4C: 0.934), CMUEcho (A2C: 0.961, A4C: 0.965); r: 0.854 (CAMUS), 0.916 (CMUEcho); ICC: 0.854 (CAMUS), 0.902 (CMUEcho) |
| Olaisen S et al[11] | 2024 | LVEF assessment (A2C and A4C, biplane Simpson's method) | U-Net | Dataset 1: 50 patients (Norway), Dataset 2: 40 patients (Norway), Dataset 3-HUNT4Echo: 2,462 patients (Norway), Dataset 4-Tromsø7: 1,032 patients (Norway) | Averaged difference, Processing time | Averaged difference: -5.5% to 0.3%; Median time: 1.9 minutes (AI-assisted) vs. 7.5 minutes (Reference) |
| Salte IM et al[12] | 2021 | GLS assessment (A2C, A3C, and A4C) | PWC-Net | 200 echocardiograms from 200 patients (Norway) | Median absolute error, MAE, Correlation coefficient, Processing time | Median absolute error: 1.4%; MAE: 1.8 %; r: 0.93; For each view processing time: 4.3 seconds, for a full patient analysis including all 3 apical views: 13.0 seconds |
| Evain E et al[13] | 2022 | GLS assessment (A2C, A3C, and A4C) | EchoPWC-Net: PWC-Net | CAMUS: 1,443 images from 30 patients (France); A clinical dataset: 1,536 images from 30 patients (France) | MAE, Correlation coefficient | MAE: 2.55% (CAMUS), 2.89% (The clinical dataset); r: 0.77 (CAMUS), 0.93 (The clinical dataset) |
| Stowell CC et al[14] | 2024 | GLS assessment (A2C, A3C, and A4C) | Unity-GLS: Higher HRnet-W32 | Development dataset (4,948 videos) (UK); External validation (300 videos from 100 patients) (UK) | Median absolute error, Correlation coefficient | Median absolute error: 1.33%; r: 0.91 |
| Holmstrøm V et al[15] | 2025 | GLS assessment (A2C, A3C, and A4C) | U-Net for segmentation; PWC-Net for motion estimation | 50 patients (Norway) | MAE, Correlation coefficient, Processing time | MAE: 1.7%; r: 0.94; Median time: 2 minutes 4 seconds (AI-assisted) vs. 4 minutes 48 seconds (Reference) |

LVEF, left ventricular ejection fraction; GLS, global longitudinal strain; LV, left ventricle; A2C, apical 2-chamber view; A3C, apical 3-chamber view; A4C, apical 4-chamber view; MAE, mean absolute error; DSC, Dice similarity coefficient; ICC, intraclass correlation coefficient; r, correlation coefficient.

4. SUPPLEMENTAL REFERENCES

1.	Mitchell C, Rahko PS, Blauwet LA, Canaday B, Finstuen JA, Foster MC, et al. Guidelines for Performing a Comprehensive Transthoracic Echocardiographic Examination in Adults: Recommendations from the American Society of Echocardiography. J Am Soc Echocardiogr. 2019;32(1). doi: 10.1016/j.echo.2018.06.004.

2.	Taub CC, Stainback RF, Abraham T, Forsha D, Garcia-Sayan E, Hill JC, et al. Guidelines for the Standardization of Adult Echocardiography Reporting: Recommendations From the American Society of Echocardiography. J Am Soc Echocardiogr. 2025;38(9):735-74. doi: 10.1016/j.echo.2025.06.001.

3.	Teed Z, Deng J. RAFT: Recurrent All-Pairs Field Transforms for Optical Flow2020 March 01, 2020:[arXiv:2003.12039 p.]. Available from: https://ui.adsabs.harvard.edu/abs/2020arXiv200312039T.

4.	Chen L-C, Zhu Y, Papandreou G, Schroff F, Adam H. Encoder-Decoder with Atrous Separable Convolution for Semantic Image Segmentation2018 February 01, 2018:[arXiv:1802.02611 p.]. Available from: https://ui.adsabs.harvard.edu/abs/2018arXiv180202611C.

5.	Chu X, Tian Z, Wang Y, Zhang B, Ren H, Wei X, et al. Twins: Revisiting the Design of Spatial Attention in Vision Transformers2021 April 01, 2021:[arXiv:2104.13840 p.]. Available from: https://ui.adsabs.harvard.edu/abs/2021arXiv210413840C.

6.	Sengupta PP, Shrestha S, Berthon B, Messas E, Donal E, Tison GH, et al. Proposed Requirements for Cardiovascular Imaging-Related Machine Learning Evaluation (PRIME): A Checklist: Reviewed by the American College of Cardiology Healthcare Innovation Council. JACC Cardiovasc Imaging. 2020;13(9):2017-35. doi: 10.1016/j.jcmg.2020.07.015.

7.	Ouyang D, He B, Ghorbani A, Yuan N, Ebinger J, Langlotz CP, et al. Video-based AI for beat-to-beat assessment of cardiac function. Nature. 2020;580(7802):252-6. doi: 10.1038/s41586-020-2145-8.

8.	Tromp J, Seekings PJ, Hung C-L, Iversen MB, Frost MJ, Ouwerkerk W, et al. Automated interpretation of systolic and diastolic function on the echocardiogram: a multicohort study. Lancet Digit Health. 2022;4(1):e46-e54. doi: 10.1016/S2589-7500(21)00235-1.

9.	Dai W, Li X, Ding X, Cheng K-T. Cyclical Self-Supervision for Semi-Supervised Ejection Fraction Prediction From Echocardiogram Videos. IEEE Trans Med Imaging. 2023;42(5):1446-61. doi: 10.1109/TMI.2022.3229136.

10.	Li H, Wang Y, Qu M, Cao P, Feng C, Yang J. EchoEFNet: Multi-task deep learning network for automatic calculation of left ventricular ejection fraction in 2D echocardiography. Comput Biol Med. 2023;156:106705. doi: 10.1016/j.compbiomed.2023.106705.

11.	Olaisen S, Smistad E, Espeland T, Hu J, Pasdeloup D, Østvik A, et al. Automatic measurements of left ventricular volumes and ejection fraction by artificial intelligence: Clinical validation in real-time and large databases. Eur Heart J Cardiovasc Imaging. 2023. doi: 10.1093/ehjci/jead280.

12.	Salte IM, Østvik A, Smistad E, Melichova D, Nguyen TM, Karlsen S, et al. Artificial Intelligence for Automatic Measurement of Left Ventricular Strain in Echocardiography. JACC Cardiovasc Imaging. 2021;14(10):1918-28. doi: 10.1016/j.jcmg.2021.04.018.

13.	Evain E, Sun Y, Faraz K, Garcia D, Saloux E, Gerber BL, et al. Motion Estimation by Deep Learning in 2D Echocardiography: Synthetic Dataset and Validation. IEEE Trans Med Imaging. 2022;41(8):1911-24. doi: 10.1109/TMI.2022.3151606.

14.	Stowell CC, Howard JP, Ng T, Cole GD, Bhattacharyya S, Sehmi J, et al. 2-Dimensional Echocardiographic Global Longitudinal Strain With Artificial Intelligence Using Open Data From a UK-Wide Collaborative. JACC Cardiovasc Imaging. 2024;17(8):865-76. doi: 10.1016/j.jcmg.2024.04.017.

15.	Holmstrøm V, Smistad E, Stølen S, Holte E, Løvstakken L, Dalen H, et al. Real-Time Global Longitudinal Strain During Echocardiography: A Deep Learning Platform for Improved Workflow. J Am Soc Echocardiogr. 2025. doi: 10.1016/j.echo.2025.08.015.
